# Mas-related G protein-coupled receptor MRGPRX2 in human basophils: Expression and functional studies

**DOI:** 10.3389/fimmu.2022.1026304

**Published:** 2023-01-16

**Authors:** Alessandro Toscano, Jessy Elst, Athina L. Van Gasse, Michiel Beyens, Marie-Line van der Poorten, Chris H. Bridts, Christel Mertens, Michel Van Houdt, Margo M. Hagendorens, Samuel Van Remoortel, Jean-Pierre Timmermans, Didier G. Ebo, Vito Sabato

**Affiliations:** ^1^ Department of Immunology, Allergology, Rheumatology and the Infla-Med Centre of Excellence, Faculty of Medicine and Health Sciences, University of Antwerp, Antwerp, Belgium; ^2^ Immunology, Allergology, Rheumatology, Antwerp University Hospital, Antwerp, Belgium; ^3^ Post-Graduate School of Allergology and Clinical Immunology, University of Milan, Milan, Italy; ^4^ Department of Pediatrics and the Infla-Med Centre of Excellence, Faculty of Medicine and Health Sciences, University of Antwerp, Antwerp, Belgium; ^5^ Pediatrics, Antwerp University Hospital, Antwerp, Belgium; ^6^ Laboratory of Cell Biology and Histology, Faculty of Pharmaceutical, Biomedical and Veterinary Sciences, University of Antwerp, Antwerp, Belgium; ^7^ Algemeen Ziekenhuis (AZ) Jan Palfijn Gent, Department of Immunology and Allergology, Ghent, Belgium

**Keywords:** basophils, allergy, CD63, CD203c, moxifloxacin, MRGPRX2, substance P.

## Abstract

**Background:**

Occupancy of MRGPRX2 heralds a new era in our understandings of immediate drug hypersensitivity reactions (IDHRs), but a constitutive expression of this receptor by basophils is debated.

**Objective:**

To explore the expression and functionality of MRGPRX2 in and on basophils.

**Methods:**

Basophils from patients with birch pollen allergy, IDHRs to moxifloxacin, and healthy controls were studied in different conditions, that is, in rest, after stimulation with anti-IgE, recombinant major birch pollen allergen (rBet v 1), moxifloxacin, fMLP, substance P (SP), or other potential basophil secretagogues. In a separate set of experiments, basophils were studied after purification and resuspension in different media.

**Results:**

Resting whole blood basophils barely express MRGPRX2 on their surface and are unresponsive to SP or moxifloxacin. However, surface MRGPRX2 is quickly upregulated upon incubation with anti-IgE or fMLP. Pre-stimulation with anti-IgE can induce a synergic effect on basophil degranulation in IgE-responsive subjects after incubation with SP or moxifloxacin, provided that basophils have been obtained from patients who experienced an IDHR to moxifloxacin. Cell purification can trigger a “spontaneous” and functional upregulation of MRGPRX2 on basophils, not seen in whole blood cells, and its surface density can be influenced by distinct culture media.

**Conclusion:**

Basophils barely express MRGPRX2 in resting conditions. However, the receptor can be quickly upregulated after stimulation with anti-IgE, fMLP, or after purification, making cells responsive to MRGPRX2 occupation. We anticipate that such “conditioned” basophils constitute a model to explore MRGPRX2 agonism or antagonism, including IDHRs originating from the occupation of this receptor.

## 1 Introduction

Human Mas-related G protein-coupled receptor member X2 (MRGPRX2) is expressed by various cell types, including dorsal root ganglion neurons and tryptase- and chymase-containing connective tissue mast cells (MC_TC_) (1), and can be activated by a variety of basic small molecules, such as the tachykinergic neuropeptide substance P (SP), anaphylatoxins, and compound 48/80, leading to degranulation independent of cross-linking of the high-affinity receptor for IgE (FcϵRI) (2, 3). Nonetheless, MRGPRX2 can also be involved in immediate hypersensitivity reactions to drugs (IDHRs), such as icatibant (4, 5), neuromuscular blocking agents (NMBAs) (4, 6–9), fluoroquinolones (4, 6–11), cetrorelix (4, 5), morphine (8, 9, 12), vancomycin (13) and many other antimicrobials/antiseptics (14, 15). However, experimental methodological heterogeneity has significantly hampered the interpretation and generalization of observations on MRGPRX2 involvement in IDHRs. This is in part due to the different experimental mutant animal models and transfected cell lines employed, both human and nonhuman, which exhibit a different level of MRGPRX2 expression, with variable receptor responsiveness and affinity (16).

In this context, a more accessible human model is cultured human mast cells from peripheral blood-progenitor cells (PBCMCs) (9), which has been successfully applied both with and without comparative silencing of MRGPRX2 (8, 17).

Two other putative and attractive candidates for further human MRGPRX2 studies were proposed by Wedi et al. (18), who showed that basophils and eosinophils constitutively express functionally active MRGPRX2 and are responsive to the fluoroquinolone ciprofloxacin. However, with respect to basophils, these data conflict with earlier preliminary findings, that is, whole blood basophils barely express MRGPRX2 (19) and basophils from uneventfully exposed control individuals do not degranulate non-specifically in response to MRGPRX2 agonists such as NMBAs, opiates, fluoroquinolones, and vancomycin (11, 20, 21). Alternatively, we demonstrated that anti-IgE and, to a lesser extent, fMLP, enhance the surface expression of MRGPRX2 by basophils (19).

Here, we investigate the discrepancies in basophilic MRGPRX2 expression and explore the effect of IgE/FcϵRI-dependent or -independent stimuli on the surface expression and functionality.

## 2 Materials and methods

### 2.1 Peripheral blood cultured mast cells (PBCMCs)

Human MC_TC_-like cells were cultured out of peripheral blood progenitor cells according to a protocol earlier described (22), and applied as a positive control for anti-MRGPRX2 staining. Briefly, CD34^+^ progenitor cells were isolated using magnetic beads (EasySep™ Human CD34 Selection Kit; Stemcell Technologies) and cultured in a serum-free methylcellulose-based medium (MethoCult SF H4236; Stemcell Technologies) supplemented with penicillin (100 units/mL), streptomycin (100 µg/mL) (Gibco, Thermo Fisher Scientific), low-density lipoprotein (10 µg/mL, LDL; Stemcell Technologies), 2-mercaptoethanol (55 µmol/L; Gibco, Thermo Fisher Scientific), stem cell factor (SCF, 100 ng/mL; Miltenyi Biotec) and interleukin-3 (IL-3, 100 ng/mL; PeproTech) during 4-5 weeks.

MCs were stained with anti-human CD117-APC (clone 104D2; BD Biosciences), and anti-human CD203c-PeCy7 (clone NP4D6; BioLegend) and defined as CD117^+^ and CD203c^+^.

The PBCMC cultures employed had a purity of 80% for CD117^+^CD203c^+^ cells (23). Before applying the PBCMCs to perform the qPCR experiments, cell debris removal was performed using the EasySep™ Dead Cell Removal (Annexin V) Kit (Stemcell Technologies).

For membrane staining of MRGPRX2, 10 µL anti-human MRGPRX2-PE (clone K125H4; BioLegend) was added before fixing the cells and incubated on ice for 20 minutes in the dark. For intracellular staining of MRGPRX2, PBCMCs were fixed with 4% paraformaldehyde (BioLegend) for 30 minutes at room temperature. Subsequently, cells were washed and permeabilized in PBS (Thermo Fisher Scientific) with 0.05% Triton-X-100 (Avantor (VWR)) (PBS-TX, pH 7.4). Then, 10 µL of anti-human MRGPRX2-PE diluted in PBS-TX was added and incubated for 20 min at 4°C. Cells were washed with 0.3 mL PBS-TX and resuspended in PBS with 0.1% sodium azide (Avantor (VWR)).

### 2.2 Peripheral whole blood basophils

Heparinized sterile whole blood samples were collected from patients allergic to birch pollen (BPAs) (all presented with rhino-conjunctivitis and/or asthma related to birch pollen exposure and documented sensitization to Bet v 1, the major allergen from *Betula verrucosa*), patients with an IDHR to moxifloxacin (MOXs) [IDHRs to moxifloxacin have been defined in a previous study (11)], and healthy control individuals (HCs) and used for immunophenotyping and activation studies.

For all the activation studies, 200 µL of whole blood were incubated with 200 µL of antigens and prewarmed at 37°C. Reactions were stopped by placing the cells on ice and adding 1 mL ice-cooled PBS-EDTA [10 mmol/L EDTA; Avantor (VWR)]. Supernatants were removed after spinning for 5 min (4°C, 200g).

Basophils were stained with 20 µL monoclonal anti-human IgE (clone GE-1; Sigma Aldrich GmBH) labeled with AlexaFluor 405 (Molecular Probes; Thermo Fisher Scientific), 10 µL monoclonal anti-human CD63-PE (clone H5C6; BD Biosciences), and 10 µL monoclonal anti-human CD203c-APC (clone NP4D6; Biolegend) and incubated on ice for 20 minutes in the dark. For membrane staining of MRGPRX2, 10 µL anti-human MRGPRX2-PE (clone K125H4; BioLegend), was added before fixing the cells and incubated on ice for 20 minutes in the dark. Cells were lysed/fixed with 2 mL BD FACS Lysing solution for 20 min at room temperature. Cells were washed twice with PBS with 0.1% sodium azide and measured. Basophils were gated as SSc^low^aIgE^+^ cells. Resting basophils were defined as CD203c^+^CD63^-^, whereas degranulating basophils were identified as CD203c^++^CD63^+^. Analyses were performed at predetermined time points.

For intracellular staining of MRGPRX2, basophils were fixed with 2 mL Phosflow Lyse/Fix Buffer (BD Biosciences) for 20 min at 37°C. Subsequently, cells were washed and permeabilized in PBS with 0.1% Triton-X-100 (PBS-TX, pH 7.4). Then, 10 µL of anti-human MRGPRX2-PE diluted in PBS-TX was added and incubated for 20 min at 4°C. Cells were washed with 0.3 mL PBS-TX and resuspended in PBS with 0.1% sodium azide.

### 2.3 Purified basophils

Basophils were isolated from EDTA-anticoagulated whole blood samples obtained from HCs using magnetic beads (EasySep™ Human Basophil Enrichment Kit; Stemcell Technologies) according to the manufacturer’s instructions and used for a different set of immunophenotyping and activation studies.

After purification, cells were resuspended in RPMI 1640 (Thermo Fisher Scientific) 10% fetal calf serum (Thermo Fisher Scientific) + gentamycin 0.5% (Thermo Fisher Scientific) + glutamine 1% (Thermo Fisher Scientific) (RPMI medium) or in Tyrode buffer 10% autologous serum (Thermo Fisher Scientific) (Tyrode medium) for 30 minutes before starting the experiments. For all the activation studies, 100 µL of purified basophils were incubated with 100 µL of antigens and prewarmed at 37°C before use. Reactions were stopped by placing the cells on ice and adding 1 mL ice-cooled PBS-EDTA (10 mmol/L EDTA). Supernatants were removed after spinning for 5 min (4°C, 200g). Basophils were stained before fixation with 20 µL monoclonal anti-human IgE (clone GE-1; Sigma Aldrich GmBH) labeled with AlexaFluor 405 (Molecular Probes; Thermo Fisher Scientific), 10 µL monoclonal anti-human CD63-PE (clone H5C6; BD Biosciences), 10 µL monoclonal anti-human CD203c-APC (clone NP4D6; Biolegend), and 10 µL anti-human MRGPRX2-PE (clone K125H4; BioLegend). Cells were lysed/fixed with 2 mL BD FACS Lysing solution for 20 min at room temperature. Cells were washed twice with PBS with 0.1% sodium azide and measured. Basophils were gated as SSc^low^aIgE^+^ cells. Resting basophils are defined as CD203c^+^CD63^-^, whereas degranulating basophils are identified as CD203c^++^CD63^+^. Analyses were performed at different predetermined time points.

### 2.4 Experiments with peripheral whole blood basophils

Basophils from BPAs and HCs were incubated with mouse anti-human monoclonal anti-IgE antibodies (10 µg/mL, clone G7-18; BD Bioscience) as a positive control or rBet v 1 (0.01 µg/mL, rBet v 1; Biomay) to assess activation/degranulation through IgE/FcϵRI cross-linking as previously described (24).

To study their IgE/FcϵRI-independent activation, basophils from BPAs and HCs were stimulated with N-formyl-methionyl-leucyl-phenylalanine (0.5 µg/mL, fMLP; Sigma-Aldrich), LPS (10 µg/mL; Sigma-Aldrich, Merck) and staphylococcus enterotoxin B (SAB 1-100 µg/mL; Sigma-Aldrich, Merck) or incubated with IL-3 (10 ng/mL; PeproTech).

To study the functionality of basophilic MRGPRX2 expression, basophils from individuals who were responsive to positive control stimulation with anti-IgE in the CD63 basophil activation test (BAT) were separately or simultaneously stimulated with anti-IgE (10 µg/mL) and the natural MRGPRX2 ligand SP (15 µmol/L, Sigma-Aldrich, Merck). MRGPRX2-mediated activation/degranulation induced by SP (1.5 µmol/L, 15 µmol/L, 150 µmol/L, and 300 µmol/L) alone or after 20 minutes of priming with IL-3 (2 ng/ml and 10 ng/ml) was also assessed.

To study moxifloxacin-induced activation/degranulation, basophils from MOXs and HCs were separately or simultaneously challenged with anti-IgE (10 µg/mL) and moxifloxacin (0.025 mmol/L or 2.5 mmol/L, Sigma-Aldrich, Merck) as previously described (11).

Whole blood basophils were also separately or jointly incubated with IL-3 (10 ng/mL), IL-33 (30 ng/mL, PeproTech), and moxifloxacin (0.025 mmol/L and 2.5 mmol/L, Sigma-Aldrich, Merck).

### 2.5 Experiments with purified basophils and preincubation with cytokines

Purified basophils were resuspended in RPMI medium or Tyrode medium and analyzed *via* a BAT. Purified basophils resuspended in the two different media were also stimulated with anti-IgE (10 µg/mL).

MRGPRX2-mediated activation/degranulation induced by SP (1.5 µmol/L, 15 µmol/L, 150 µmol/L, and 300 µmol/L) alone or after 20 minutes of priming with IL-3 (2 ng/ml and 10 ng/ml) was assessed in purified basophils resuspended in RPMI medium.

These were also separately or jointly incubated with IL-3 (10 ng/mL), IL-33 (30 ng/mL), and moxifloxacin (2.5 mmol/L).

### 2.6 Flow cytometric analysis

Flow cytometric analysis was performed on a FACSCanto II™ flow cytometer (BD Immunocytometry Systems) equipped with three lasers (405, 488, and 633 nm). Correct compensation settings for the antibodies conjugated with fluorochromes were performed using BD CompBeads (BD Biosciences). Fluorescence minus one (FMO) samples were used to set a marker for positivity according to the 99th percentile. Flow cytometric data were analyzed using Kaluza Analysis 2.1 software (Beckman Coulter). Flow cytometric characterization of basophils relied upon a combination of side scatter (SSC), anti-IgE and CD203c. At least 1,000 basophils were counted and analyzed. Activation is expressed in net percentages of upregulation of CD63, CD203c, and MRGPRX2, that is the percentage of CD63, CD203c, and MRGPRX2 positive stimulated cells minus the percentage of positive CD63, CD203c, and MRGPRX2 resting cells.

### 2.7 RT-qPCR and gel electrophoresis

RNA isolation from PBCMCs and purified basophils at rest or after stimulation with anti-IgE (10 µg/mL) from 3 HCs was performed using the Nucleospin RNA XS kit, according to the manufacturer’s protocol (Macherey-Nagel). Sample RNA concentration and quality were determined using the Agilent Bioanalyzer 2100 platform (Agilent Tech.). An RNA integrity number (RIN) cut-off of 6 was applied to exclude inadequate samples. A total of 200 ng RNA was reverse-transcribed using the iScript cDNA synthesis kit (Bio-Rad Laboratories, Hercules), and the resulting cDNA was diluted 1:5. Bio-Rad PrimePCR assays containing validated primer pairs were used to perform a qPCR analysis on the expression of the target gene MRGPRX2 (PrimePCR Assay ID qHsaCID0023564), and two housekeeping genes, namely HPRT1(PrimePCR Assay ID qHsaCID0016375) and RPS29 (PrimePCR Assay ID qHsaCED0038808) (25), applied as positive controls. Each sample was run in triplicate.

RT-qPCR was performed on 2 µL of cDNA using the SSO Advanced Universal SYBR Green Supermix (Bio-Rad Laboratories), with a total of 40 amplification cycles and the PCR protocol according to the manufacturer’s instructions. Next, qPCR products underwent 2% agarose gel electrophoresis and UV visualization (GelRed Nucleic acid stain, Biotium) to evaluate the presence of amplicons of the expected band size.

### 2.8 Statistical analysis

Two-way analysis of variance (ANOVA), paired Student’s t-tests, Tukey’s multiple comparisons test, Mann Whitney test and Pearson’s correlation coefficient were applied, where appropriate, using JMP Pro 13 (SAS, Cary, NC, USA). P-values < 0.05 were considered as significant. Figures were developed in GraphPad Prism 7 (GraphPad Software, La Jolla, CA, USA).

## 3 Results

### 3.1 MRGPRX2 expression in resting basophils and PBCMCs

**Figure 1:**
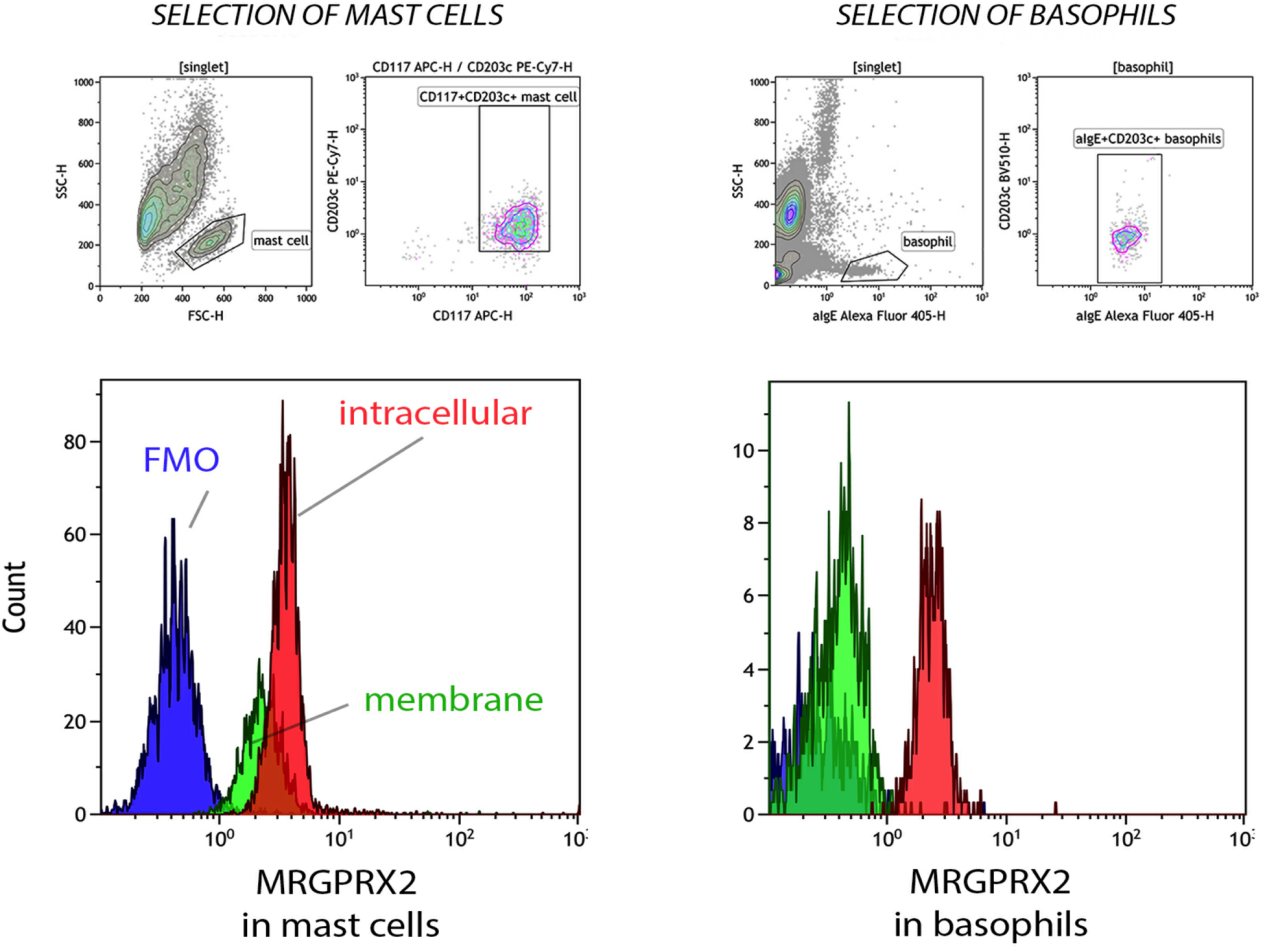
Membrane and intracellular expression of MRGPRX2 in peripheral blood cultured mast cells (PBCMCs) and whole blood basophils. Representative plots of membrane (green histogram) and intracellular (red histogram) MRGPRX2 expression in PBCMCs and resting whole blood basophils. FMO = fluorescence minus one sample (blue histogram). PBCMCs = peripheral blood cultured mast cells.

Resting PBCMCs express MRGPRX2, both intracellularly and on their surface membrane (**Figure 1**). In contrast, resting whole blood basophils from BPAs and HCs invariantly show intracellular staining for MRGPRX2, but barely express the receptor on their surface membrane.

Whereas MRGPRX2 mRNA is detected in PBCMCs by qPCR, it was not found in resting purified basophils at rest or after stimulation with anti-IgE, as shown after gel electrophoresis of the qPCR products (**Supplementary Figures 1, 2**).

For both housekeeping genes (HPRT1 and RPS29) qPCR was successful and showed clear expression in all samples indicating that positive control genes are clearly expressed in both PBCMCs and resting or anti-IgE-stimulated purified basophils.

### 3.2 MRGPRX2, CD63, and CD203c expression by activated whole blood basophils

**Figure 2:**
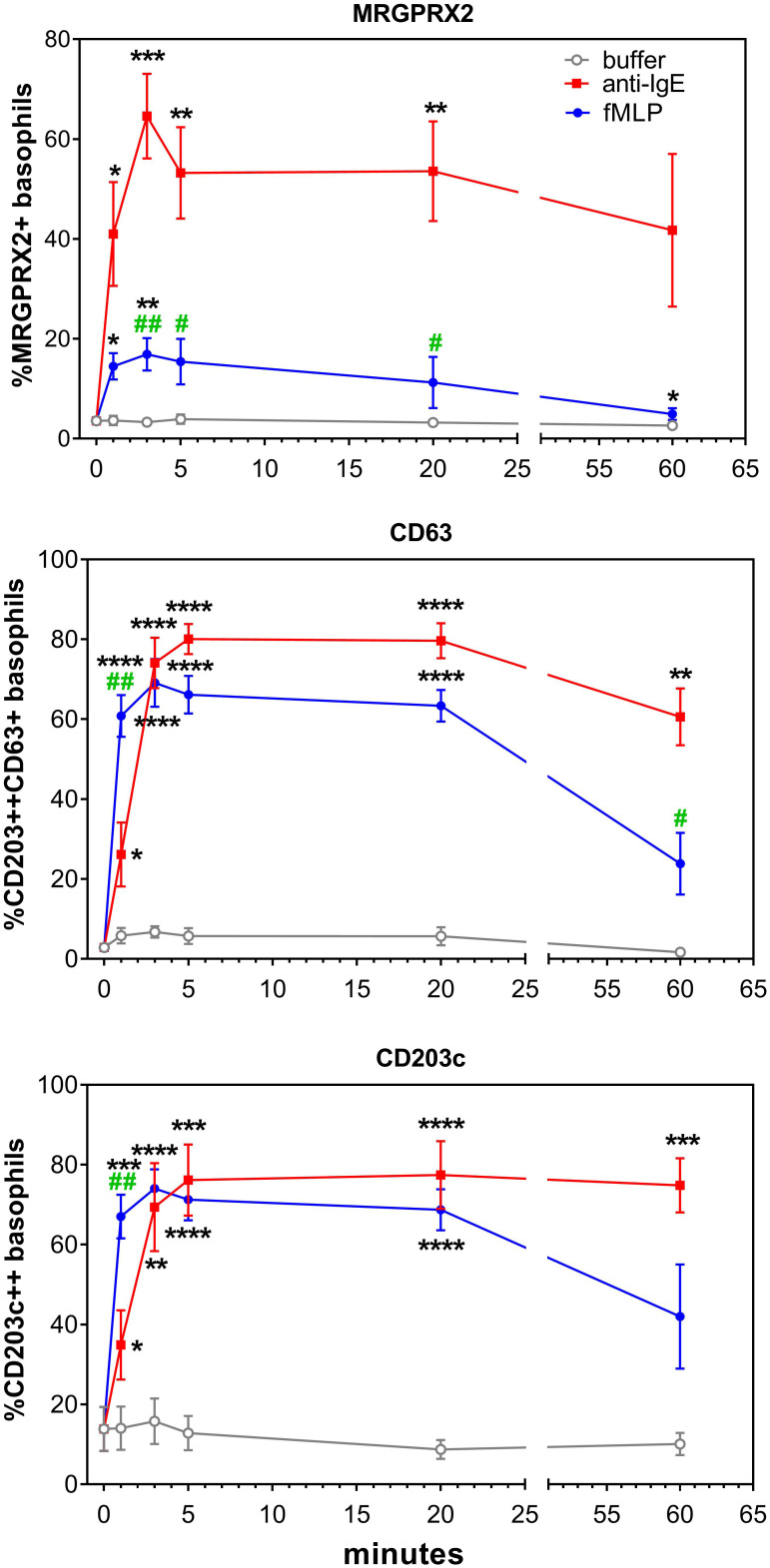
Effect of anti-IgE and fMLP stimulation on membrane MRGPRX2 expression in whole blood basophils. **(A)** Representative plots on the effect of anti-IgE and fMLP stimulation of 20 min on membrane MRGPRX2 expression of whole blood basophils from one healthy control. Resting cells are identified as IgE^+^CD203c^+^CD63^-^ (green). MRGPRX2^+^ expressing cells are indicated in purple. **(B)** Time kinetics of MRGPRX2, CD63 and CD203c membrane expression of whole blood basophils after stimulation with anti-IgE (red), fMLP (blue) or buffer (grey) (n=8). * p<0.05; **p<0.01 ***p<0.001; ****p<0.0001 compared to the buffer. ^#^p<0.05; ^##^p<0.01 compared to the anti-IgE stimulation; Tukey’s multiple comparisons tests.

Stimulation with anti-IgE and fMLP induces an upregulation of MRGPRX2. This significant upregulation peaks after 3 minutes and reaches a plateau at 60 minutes. The appearance of the degranulation marker CD63 and upregulation of CD203c display slightly dissimilar time kinetics, since both events peak later, i.e., after 5 minutes (**Figure 2**). MRGPRX2 upregulation after stimulation with fMLP is significantly less pronounced compared to anti-IgE stimulation.

**Figure 3:**
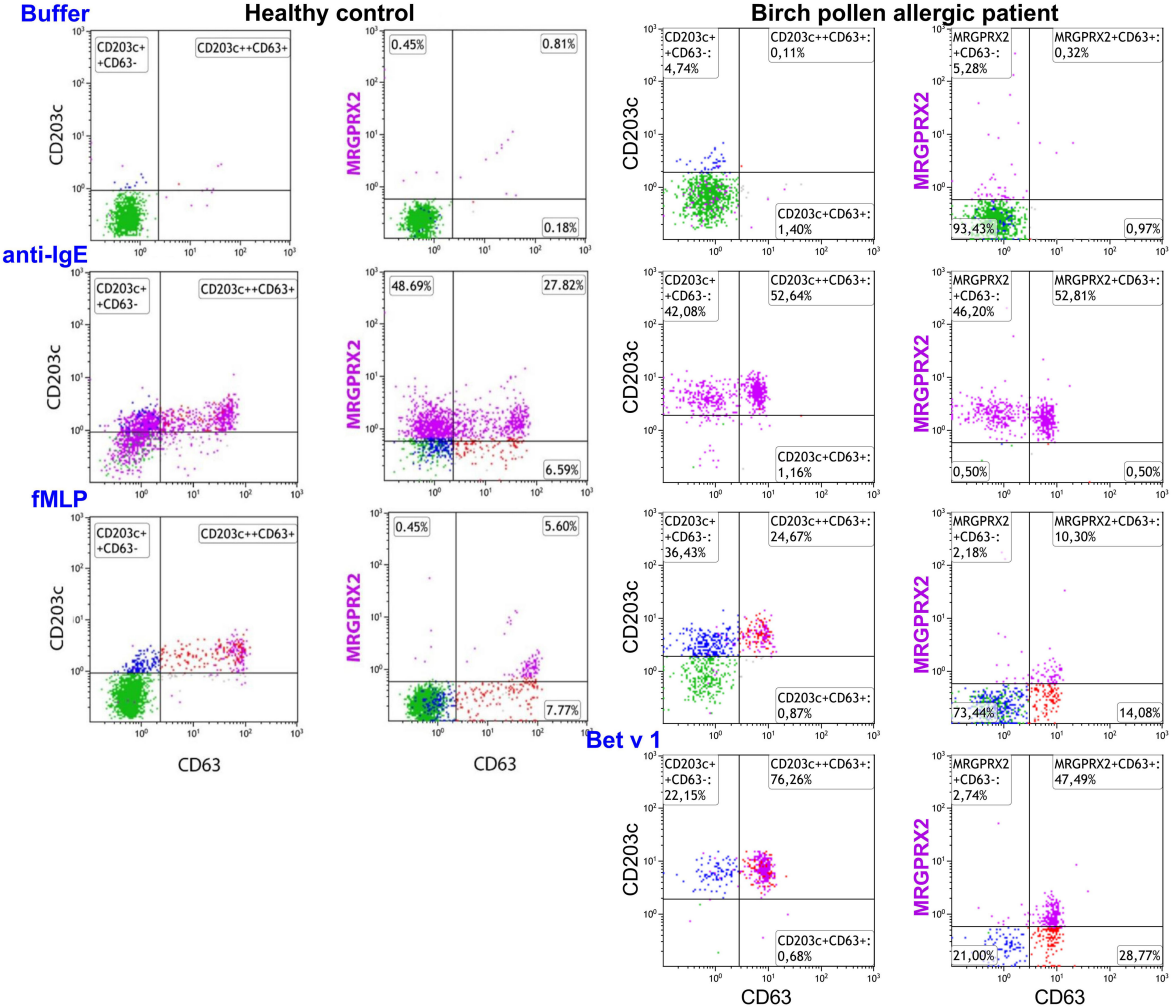
Effect of anti-IgE and fMLP stimulation on membrane MRGPRX2 expression in birch allergic patients. Representative plots on the effect of anti-IgE, fMLP or Bet v 1 stimulation of 20 min on membrane MRGPRX2 expression of whole blood basophils from one birch pollen allergic individual. Resting cells are identified as IgE^+^CD203c^+^CD63^-^ (green). MRGPRX2^+^ expressing cells are indicated in purple.

Representative plots of MRGPRX2 upregulation in HC and BPA after 20 minutes stimulation with anti-IgE, fMLP, and Bet v 1 are shown in **Figure 3**.

No difference in MRGPRX2 expression was observed between resting basophils from BPAs (n=16; median 4%; range 0-14%) and HCs (n=10; median 6%; range 4-20%). On the other hand, a significantly higher MRGPRX2 upregulation was observed in anti-IgE stimulated basophils from BPAs (n=16; median 50%; range 13-99%) when compared to HCs (n=10; median 19%; range 12-31%) (*p* = 0.02, Mann Whitney test).

IL-3 triggers upregulation of CD203c without any upregulation of MRGPRX2 or CD63 (**Supplementary Figure 3**). We could not detect any upregulation of CD203c, CD63 or MRGPRX2 after stimulation with LPS and Staphylococcus enterotoxin (data not shown).

### 3.3 Co-incubation experiments with anti-IgE and substance P

**Figure 4:**
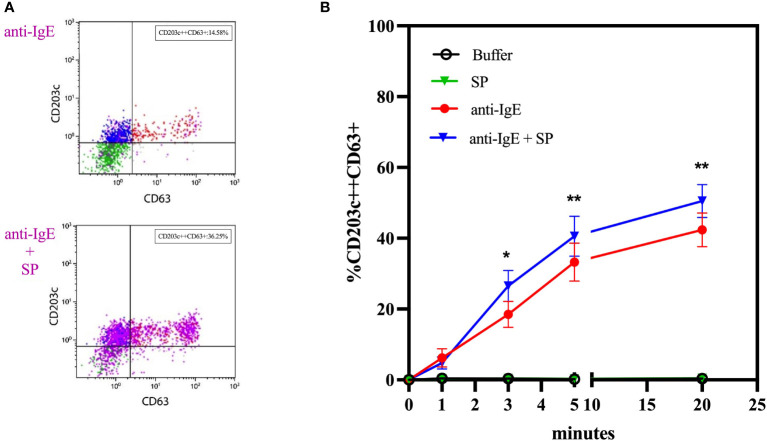
Co-incubation experiments with anti-IgE and substance P. **(A)** Representative plots of the effect of co-incubation of anti-IgE and SP on the membrane expression of CD63 of whole blood basophils in comparison to anti-IgE alone. **(B)** Time kinetics of the effect of SP, anti-IgE and SP in co-incubation with anti-IgE on the membrane expression of CD63 of whole blood basophils (n=10). SP = substance P. * *p* ≤ 0.05; ** *p* ≤ 0.01 anti-IgE compared to anti-IgE + SP at each time point; paired Student’s t-tests.

In whole blood basophils from individuals who are responsive to anti-IgE, SP alone does not induce upregulation of CD63, CD203c, or MRGPRX2 surface expression. In contrast, co-incubation with anti-IgE and SP exerts a numeric synergistic effect on CD63 upregulation reaching significance after 3 minutes (*p* = 0.0104 at 3 minutes; *p* = 0.0017 at 5 minutes; *p* = 0.0026 at 20 minutes) (**Figure 4**).

### 3.4 Co-incubation experiments with anti-IgE and moxifloxacin

**Figure 5:**
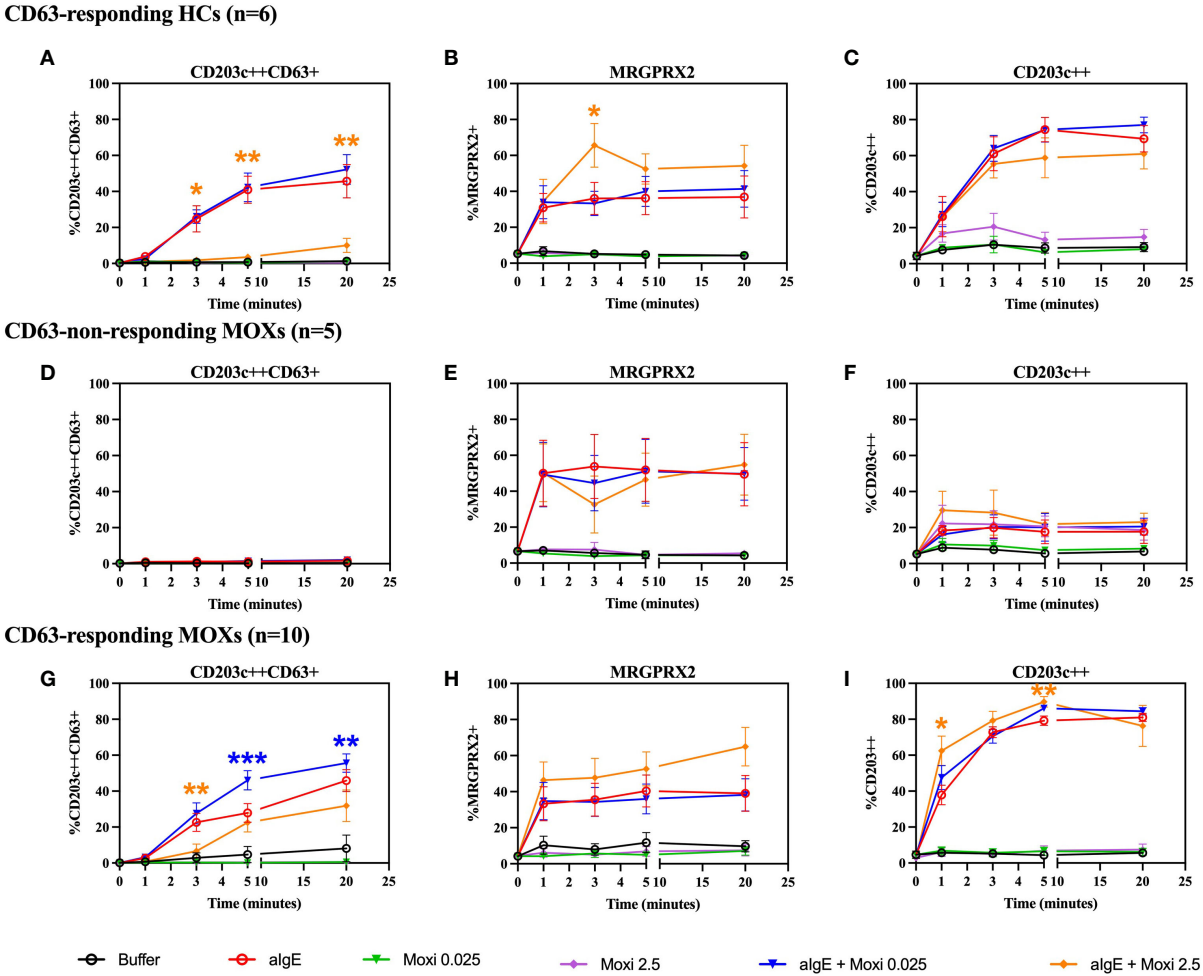
Time kinetics of co-incubation experiments with anti-IgE and moxifloxacin. Whole blood basophils are stimulated with anti-IgE and moxifloxacin (0.025 mmol/L and 2.5 mmol/L) alone or in co-incubation with anti-IgE. The figure shows the effect on CD63, MRGPRX2 and CD203c membrane expression from CD63-responding HCs (n=6) **(A-C)**, CD63-non-responding MOXs (n=5) **(D-F)** and CD63-responding MOXs (n=10) **(G-I)**. For experiments with moxifloxacin 2.5 mmol/L, alone or in coincubation with anti-IgE, in CD63-responding MOXs, n=7. aIgE=anti-IgE; Moxi: moxifloxacin; MOXs = patients with immediate type hypersensitivity to moxifloxacin; HCs = healthy controls. Blue asterisks: statistical significance of aIgE + Moxi 0.025 mmol/L compared to aIgE; orange asterisks: statistical significance of aIgE + Moxi 2.5 mmol/L compared to aIgE; * *p* ≤ 0.05; ** *p* ≤ 0.01; *** *p* ≤ 0.001; paired Student’s t-tests.

Based on the appearance of CD63, stimulation with anti-IgE reveals two distinct basophil reactivity patterns, i.e., “CD63 responders” and “CD63 non-responders”. HCs and CD63-responding MOXs show an anti-IgE-induced appearance of CD63 (**Figures 5A, G**) and upregulation of surface MRGPRX2 (**Figures 5B, H**). CD63-non-responding MOXs fail to demonstrate an increase of CD63 expression (**Figure 5D**) but also show surface upregulation of MRGPRX2 (**Figure 5E**). In all three groups, upregulation of CD203c is observed (**Figures 5C, F, I**).

Moxifloxacin alone does not induce degranulation with the appearance of CD63 or surface upregulation of MRGPRX2 in either MOXs, regardless of CD63-responding status, (**Figures 5D, E, G, H**) or HCs (**Figures 5A, B**).

Like for SP, co-incubation of the cells with anti-IgE and moxifloxacin 0.025 mmol/L exerts a synergistic effect with enhanced upregulation of CD63, which is strictly restricted to the CD63-responding MOXs and reaches statistical significance from 5 minutes onwards (*p* = 0.0007 at 5 minutes; *p* = 0.004 at 20 minutes) (**Figure 5G**). In contrast, in CD63-non-responding MOXs (**Figure 5D**) and in responsive HCs (**Figure 5A**), co-incubation with anti-IgE and moxifloxacin 0.025 mmol/L does not result in a CD63 expression higher than with anti-IgE alone. No significant difference is observed in all three groups on the expression of MRGPRX2 (**Figures 5B, E, H**) and CD203c (**Figures 5C, F, I**) in comparison to experiments with anti-IgE alone. A representative plot is shown in **Supplementary Figure 4**.

In contrast, coincubation with anti-IgE and moxifloxacin 2.5 mmol/L seems to have an antagonistic effect on anti-IgE-mediated CD63 upregulation at 3 minutes in CD63-responding MOXs (*p* = 0.005), but not in CD63-non-responding MOXS, and from 3 minutes onwards (*p* = 0.0241 at 3 minutes; *p* = 0.0026 at 5 minutes; *p* = 0.006 at 20 minutes) in responsive HCs (**Figures 5A, D, G**). Interestingly, the magnitude of inhibition seems to be higher in HCs than in CD63-responding MOXs. Furthermore, coincubation with anti-IgE and moxifloxacin 2.5 mmol/L has a synergistic effect on MRGPRX2 upregulation at 3 minutes only in responsive HCs (*p* = 0.0256) (**Figure 5B**). No correlation between the magnitude of the inhibitory effect of coincubation with moxifloxacin 2.5 mmol/L on anti-IgE-mediated CD63-upregulation and MRGPRX2 upregulation was observed for any subpopulation (**Supplementary Figure 5**). With respect to CD203c, coincubation with anti-IgE and moxifloxacin 2.5 mmol/L triggers significant synergistic upregulation only in CD63-responding MOXs (*p* = 0.0019 at 1 minute; *p* = 0.0039 at 5 minutes) (**Figure 5I**).

### 3.5 MRGPRX2 expression in purified basophils

**Figure 6:**
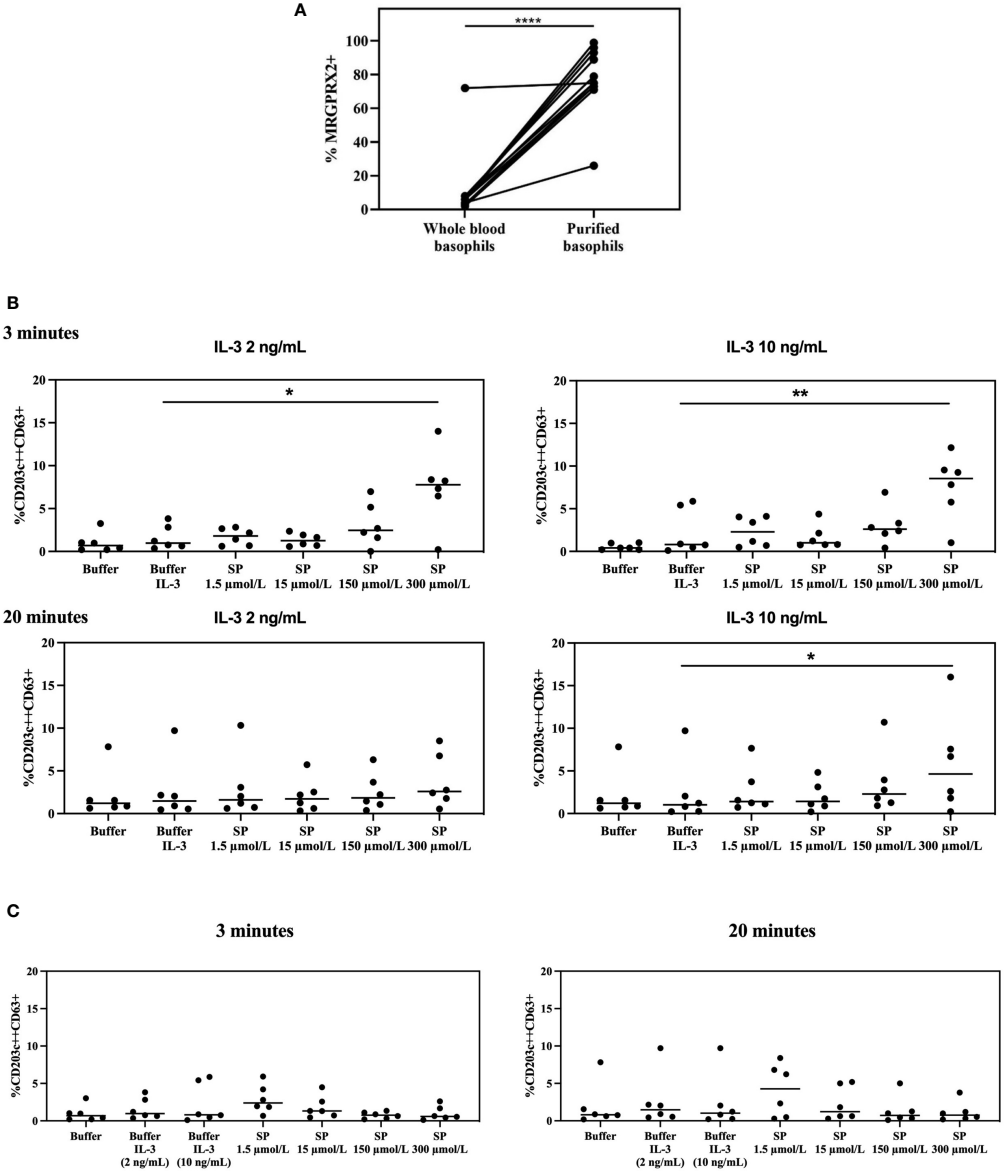
MRGPRX2 expression in whole blood vs purified basophils and MRGPRX2 functionality in purified basophils. **(A)** Effect on MRGPRX2 expression on the surface membrane of basophils from HCs after purification using magnetic beads (n=12). **** *p* ≤ 0.0001; paired Student’s t-tests. **(B)** Membrane expression of CD63 on purified basophils from HCs resuspended in RPMI medium, primed with IL-3 (2 ng/mL and 10 ng/mL) and subsequently stimulated with SP for 3 and 20 minutes (n=6). * *p* ≤ 0.05; ** *p* ≤ 0.01 SP 300 µmol/L after priming with IL-3 compared to buffer after priming with IL-3; paired Student’s t-tests. **(C)** Membrane expression of CD63 on purified basophils from the same HCs as in Figure 6B resuspended in RPMI medium, without priming with IL-3, and stimulated with SP for 3 and 20 minutes (n=6). HCs = healthy controls. SP: substance P.

As shown in **Figure 6A** and **Supplementary Figure 5**, purification of basophils induces a significantly higher MRGPRX2 expression as compared to whole blood basophils (*p* ≤ 0.0001).

Purified basophils, resuspended in both Tyrode medium and RPMI medium, show upregulation of CD203c but not CD63. Purified basophils incubated in RPMI medium are no longer stimulable with anti-IgE and their MRGPRX2 density is higher than that observed for separated basophils suspended in Tyrode medium (**Supplementary Figure 7**).

### 3.6 MRGPRX2 functionality in purified basophils

Stimulation of purified basophils resuspended in RPMI medium with SP, primed with two different concentrations of IL-3 for 20 minutes, induces upregulation of CD63 after 3 minutes with a direct SP-dependent dose effect that reaches significance for the highest tested concentration of SP (*p* = 0.0275 for SP 300 µmol/L after priming with IL-3 2 ng/mL compared to buffer after priming with IL-3 2 ng/ml; *p* = 0.0042 for SP 300 µmol/L after priming with IL-3 10 ng/mL compared to buffer after priming with IL-3 10 ng/mL). At 20 minutes the effect is significant only for experiments conducted jointly with the highest IL-3 and SP concentrations (*p* = 0.0394 for SP 300 µmol/L after priming with IL-3 10 ng/ml compared to buffer after priming with IL-3 10 ng/mL) (**Figure 6B**).

No significant SP-induced CD63 upregulation is observed in the same set of experiments performed without co-incubation with IL-3 or on whole-blood basophils (**Figure 6C** and **Supplementary Figure 8**).

Priming with IL-3 has no effect on MRGPRX2 expression, neither on purified nor on whole blood basophils (**Supplementary Figure 9**).

### 3.7 Moxifloxacin-induced degranulation of purified basophils

**Figure 7:**
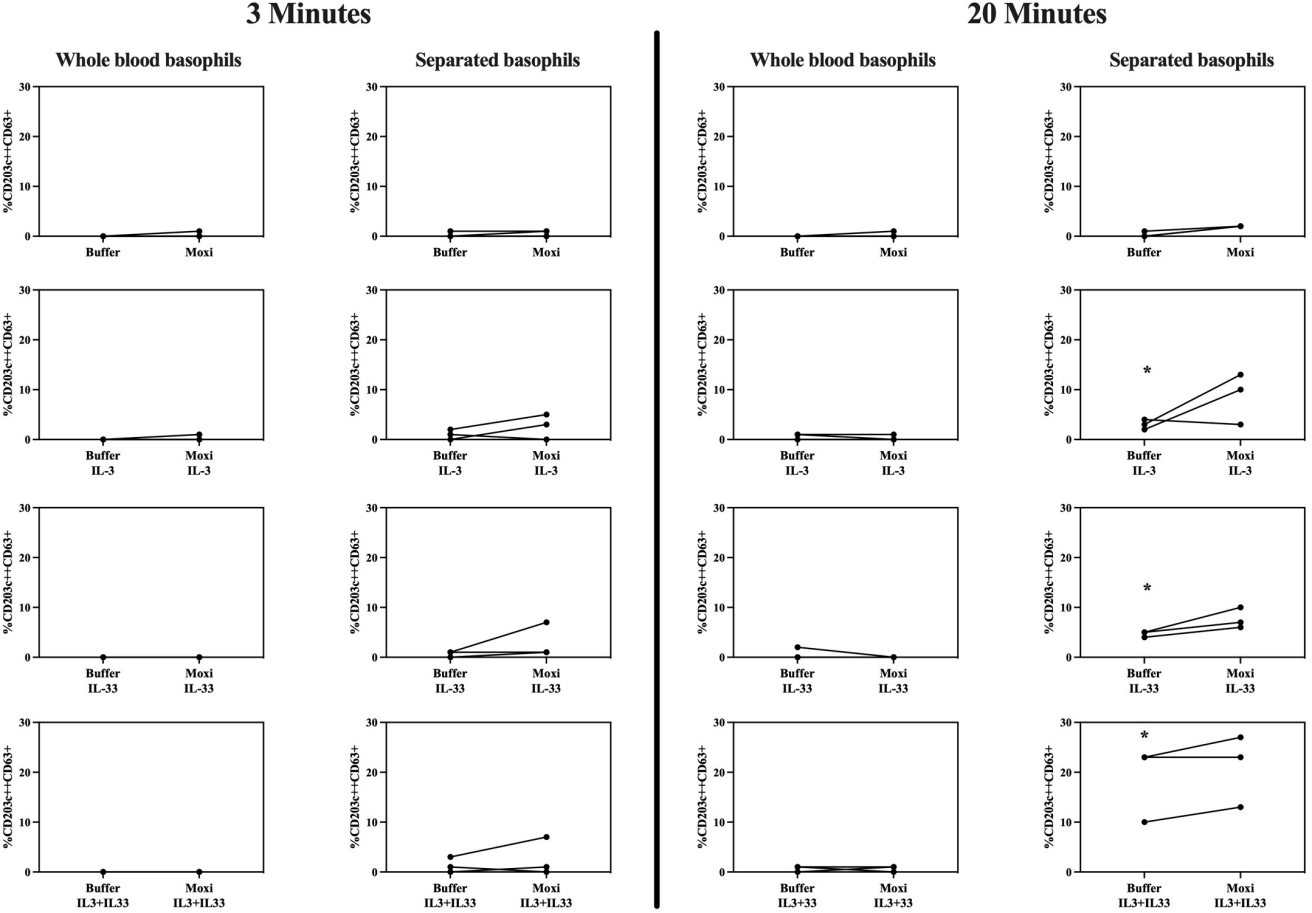
Stimulation with moxifloxacin of whole blood basophils vs purified basophils. Membrane expression of CD63 of whole blood basophils and purified basophils resuspended in RPMI medium from 3 HCs after incubation with IL-3 (10 ng/mL), IL-33 (30 ng/mL), and moxifloxacin (2.5 mmol/L) alone or in co-incubation with IL-3 (10 ng/mL) and/or IL-33 (30 ng/mL) (analyses performed at 3 and 20 minutes) (n=3). HCs = healthy controls. * *p* ≤ 0.05 compared to buffer; paired Student’s t-tests.

As shown in **Figure 7**, stimulation of whole blood basophils or purified basophils resuspended in RPMI medium with moxifloxacin alone results in a CD63 expression comparable to spontaneous expression of CD63.

IL-3 and IL-33 separately can significantly (but slightly) increase CD63 expression in purified basophils at 20 minutes with a (not significant) synergistic effect when combined. In purified basophils, co-incubation of IL-3 and/or IL-33 with moxifloxacin seems to cause an additive effect on CD63 upregulation which is not statistically significant.

Almost no upregulation of CD63 is observed in whole blood basophils obtained from the same patients, regardless of incubation with IL-3, IL-33, and/or moxifloxacin.

Co-incubation of both purified and whole blood basophils with IL-3, IL-33, or both, fails to induce upregulation of MRGPRX2 after 3 and 20 minutes (**Supplementary Figure 10**).

## 4 Discussion

Here we confirm that human resting peripheral blood basophils only rarely express functionally active MRGPRX2 on their surface membrane and, consequently, are unresponsive to endogenous and exogenous MRGPRX2 agonists. Although unclear, this probably correlates with the lack of exposure to specific tissue factors and allows the prevention of potent and potentially harmful nonspecific activation of these cells, as MRGPRX2 can also be activated by various exogenous and endogenous substances including serum albumin fragments (26).

Our data are not in line with the observations by Wedi et al. (18), who demonstrated that MRGPRX2 is constitutively expressed on resting isolated basophils, ciprofloxacin induces basophil degranulation, and basophils contain MRGPRX2 mRNA. Net of methodological differences, our study confirms that basophils express MRGPRX2. However, according to our results, extracellular expression of the receptor and its engagement by endogenous and exogenous ligands seems to require a previous conditioning (that can also be elicited by the experimental conditions themselves). In fact, constitutive surface expression of MRGPRX2 by basophils would be difficult to align with previous results on moxifloxacin, other fluoroquinolones, opioids, and neuromuscular blocking agents, all known MRGPRX2 agonists (11, 20, 21, 27–32).

In support of our hypothesis, we show that cell purification with magnetic beads actively induces surface expression of MRGPRX2 in basophils. Furthermore, purified basophils resuspended in the same culture medium as used by Wedi et al. (18) showed a higher density of MRGPRX2 than those resuspended in a different solution and became unresponsive to an IgE-mediated stimulus. This might imply that this specific culture medium may further enhance the expression of MRGPRX2. However, even after purification and resuspension in the same culture medium as that used by Wedi et al. (18), the basophils in our experiments did not exhibit spontaneous surface upregulation of CD63 and were still unable to degranulate after stimulation with an MRGPRX2 agonist. Indeed, further conditioning with IL-3 (and/or IL-33) was necessary to achieve significant degranulation with substance P or moxifloxacin, but neither IL-3 nor IL-33 was responsible for an increase in MRGPRX2 expression that might have underpinned such basophil activation.

Overall, some subtle differences in the two experimental settings may have played a role in these discrepancies. However, as demonstrated by the series of experiments performed with substance P and moxifloxacin with whole blood basophils versus purified basophils from healthy donors, MRGPRX2 ligands appear to be able to selectively induce degranulation in purified basophils. This suggests that cell purification is responsible for the expression of functional MRGPRX2.

Unlike Wedi et al, but in agreement with the data from the FANTOM 5 project (33), we could not demonstrate the presence of MRGPRX2 mRNA in resting basophils. The reason for these conflicting findings is unclear. The observation that the levels of mRNA and protein are poorly correlated is not an infrequent phenomenon. For example, it has been shown that the expression of FcϵRI does not necessitate large numbers of mRNA molecules (34). The same could also be true for MRPGRX2 in basophils.

Alternatively, MRGPRX2 is confirmed to be ubiquitously and abundantly expressed intracellularly in resting whole blood basophils and surface expression is rapidly upregulated in response to IgE/FcϵRI-dependent and IgE/FcϵRI-independent activation of the cells. At present, the exact intracellular localization of MRGPRX2 in basophils remains unknown. Its upregulation displays different time kinetics and magnitudes depending on the activation mode. All three readings, i.e., the ectoenzyme CD203c, the lysosomal degranulation marker CD63, and MRGPRX2, reveal fMLP, which acts *via* FPR-1, another G-protein-coupled receptor (GPCR) (35), to trigger a faster but transient and significantly less pronounced activation of the cells as compared to cross-linking of IgE/FcϵRI by anti-IgE or a relevant allergen (e.g., rBet v 1 in BPAs). These data parallel the findings by Knol et al. (36), who showed basophilic histamine release by IgE/FcϵRI cross-linking to be slower than the almost instantaneous release in response to fMLP and the observations in MCs by Gaudenzio et al. (5), who demonstrated that IgE-independent activation triggers a more rapid but transient degranulation as compared to IgE/FcϵRI cross-linking. Furthermore, priming with IL-3 selectively induces CD203c upregulation without upregulation of CD63 or MRGPRX2 and, in CD63-non-responding moxifloxacin hypersensitive patients, MRGPRX2 upregulation occurs independently of CD63 appearance. Collectively these data suggest that MRGPRX2 is localized in a third intracellular compartment.

Furthermore, our experiments show that basophilic expression of MRGPRX2 could also contribute to pathological conditions such as IDHRs resulting from the off-target occupation of non-immune receptors. At present, most studies on the ability of drugs to activate MRGPRX2 have been conducted with murine MCs (4), transfected HEK cells (4), and human MC lines (e.g., LAD2) (4, 6, 12), or *in vitro* CD34^+^-derived human MCs (5, 7–9, 22). Based on these studies it has been proposed that the occupation of MRGPRX2 could be responsible for IDHRs to several drugs and that the murine orthologue MrgprB2 might serve as a model for the development of therapeutic strategies aimed at preventing or treating a subset of IDHR. However, a comparison of the data of McNeil et al. (4) with the findings by Azimi et al. (6) and our own observations (8), reveals significant species-specific differences, which might hinder the translation of findings in mice to humans making this model not suitable for the development of therapeutic strategies. Alternatively, the LAD2 cells have been shown to be intermediately differentiated as compared to human mature skin MCs and to variably express MRGPRX2 (37).

Based on our data, we anticipate that “conditioned” basophils could serve as a human model to explore IDHRs resulting from the MRGPRX2 occupation. Moreover, as such MRGPRX2-dependent IDHRs only occur in a minority of exposed individuals, and do not necessarily involve all drugs with MRGPRX2-agonistic properties, our approach using “conditioned” patients’ basophils could allow capturing data that are inaccessible when using animal models or techniques based upon cell lines or healthy donor cells. The reason why not all individuals exposed to a substance capable of activating MRGPRX2 react with an IDHR has not yet been elucidated with certainty but is probably attributable to polymorphisms in the receptor. For instance, mutations in the carboxyl terminus of MRGPRX2, the portion responsible for phosphorylation and desensitization of the receptor, can make mast cells more responsive to ligands such as SP (38). Admittedly, using humanized cell cultures expressing a specific variant of MRGPRX2, while not equally easily accessible, could be another possibility for an individualized study of the receptor.

In this context, we show that in patients with immediate hypersensitivity to moxifloxacin, co-incubation of the basophils with anti-IgE and moxifloxacin induces a more pronounced degranulation as compared to IgE/FcϵRI cross-linking by anti-IgE alone. This is in accordance with the recent findings that IgE-mediated and MRGPRX2 activation can synergistically combine to boost the exocytosis of cutaneous MCs (39).

Remarkably, even though IgE-mediated upregulation of MRGPRX2 is observed in all the subjects, synergistic degranulation is restricted to patients with immediate-type hypersensitivity to moxifloxacin with a “CD63-responder” status of the basophils. The reasons why patients with a “CD63-non-responder status” and controls with a “CD63-responder status” do not show this synergistic effect remain elusive. To some extent, the different behavior between CD63-responding patients and CD63-responding controls could relate to polymorphisms, mutations, and epigenetic modifications affecting MRGPRX2-driven signaling (40–42).

Similarly unclear, there seems to be a conundrum with co-stimulation with anti-IgE and moxifloxacin, with opposite findings for low and high stimulation concentrations of this drug. The reasons for the antagonistic effect of the highest tested concentration of moxifloxacin on anti-IgE induced degranulation observed in CD63-responders, regardless of their clinical status, remain elusive. Babina et al. recently described a synergistic effect on the degranulation of mast cells for low concentrations of two different MRGPRX2 ligands, namely SP and codeine. Of note, for higher concentrations of the same ligands, no agonistic effect on the degranulation of one stimulus on the other was observed (39). Whether extremely high concentrations of MRGPRX2 ligands could lead to the initiation of a counterregulatory mechanism, which in our case may have influenced IgE-mediated degranulation, remains speculative. The existence of extensive crosstalk between IgE-mediated and non-IgE-mediated pathways and their intracellular signaling has recently been described in detail (43). For instance, despite the existence of a redundant and overlapping signaling network between the two pathways, calcium channels differentially affect PI3K activation in FcϵRI- compared to MRGPRX2-mediated signaling, which is a crucial intracellular signal transducer for both (44). This could result in a counterregulatory mechanism that avoids noxious degranulation (43). Particularly interesting is that while in HCs there is no synergistic effect of moxifloxacin 0.025 mmol/L, moxifloxacin 2.5 mmol/L still manages to have an inhibitory effect on anti-IgE-mediated degranulation; moreover, this effect is visibly higher than that seen in CD63-responding patients. One could therefore speculate that high concentrations of moxifloxacin succeed in stimulating MRGPRX2 in these healthy subjects without causing degranulation, but with an exclusive counterregulatory effect, whereas a full degranulation effect is already observed at lower concentrations in patients. It might be precisely the result of residual MRGPRX2-mediated degranulation that accounts for the lower inhibition observed in CD63-responding patients. Not to be overlooked, a direct pharmacological effect of high concentrations of moxifloxacin on calcium channels may have led to this effect, because of their key role and differential effects on IgE- and non-IgE-mediated pathways. Fluoroquinolones can interact directly with calcium channels. In fact, they induce a multi-ion channel–blocking action in the heart within the supra-therapeutic dose range and can exert insulin secretion *via* the Ryanodine receptor activation and the active influx of calcium from the extracellular space in pancreatic β-cells (45, 46). Clearly, MRGPRX2 signaling remains unclear and definitely is more than currently meets the eye.

Admittedly, our *ex vivo* model of co-incubation with anti-IgE and moxifloxacin does not exactly mirror *in vivo* conditioning of the cells during infection/inflammation. However, for the time being, except for fMLP and rBet v 1 in patients with birch pollen allergy, we failed to identify other substances that promote surface upregulation of MRGPRX2 by basophils. Neither LPS nor Staphylococcus enterotoxin seems to have any effect. Similarly, no significant upregulation of MRGPRX2 could be induced in MCs either, despite numerous efforts (47). Wedi et al. described a dose-dependent increase of MRGPRX2 surface expression in purified basophils after 30 minutes of incubation with IL-3, a well-established primer of basophils (48). Upregulation of the receptor was observed also after 24 hours of incubation with IL-3, anti-IgE, C5a, or fMLP (18). However, we failed to observe an IL-3 induced upregulation of MRGPRX2, neither in whole blood nor in purified basophils.

In conclusion, we show that circulating basophils can be rapidly “conditioned” to respond to the occupation of *de novo* MRGPRX2 surface expression. Moreover, since resting basophils of uneventfully exposed control individuals do not respond non-specifically to drugs requiring MRGPRX2 involvement (11, 20, 21, 28–32), it is tempting to hypothesize that comparative studies with and without “conditioned” cells might enable discrimination between IDHRs from genuine cross-linking of IgE/FcϵRI and MRGPRX2 occupation.

## Data availability statement

The raw data supporting the conclusions of this article will be made available by the authors, without undue reservation.

## Ethics statement

The studies involving human participants were reviewed and approved by Ethics committee of the Antwerp University Hospital. The patients/participants provided their written informed consent to participate in this study.

## Author contributions

VS designed the study. AV, MB, and M-LvP enrolled the patients. CM, who also contributed to the experimental design, performed the experiments with CB and MVH. The experiments with the polymerases chain reaction were performed by SVR and J-PT. AT and JE performed the data analysis and wrote the first draft of the paper. DE, MH, and VS coordinated and supervised the project and contributed to writing the paper. All authors contributed to the article and approved the submitted version.

## References

[1] Varricchi G Pecoraro A Loffredo S Poto R Rivellese F Genovese A . Heterogeneity of human mast cells with respect to MRGPRX2 receptor expression and function. Front Cell Neurosci (2019) 13:299. doi: 10.3389/fncel.2019.00299 31333418 PMC6616107

[2] Subramanian H Kashem SW Collington SJ Qu H Lambris JD Ali H . PMX-53 as a dual CD88 antagonist and an agonist for mas-related gene 2 (MrgX2) in human mast cells. Mol Pharmacol (2011) 79:1005–13. doi: 10.1124/mol.111.071472 PMC3102546 21441599

[3] Kashem SW Subramanian H Collington SJ Magotti P Lambris JD Ali H . G Protein coupled receptor specificity for C3a and compound 48/80-induced degranulation in human mast cells: Roles of mas-related genes MrgX1 and MrgX2. Eur J Pharmacol (2011) 668:299–304. doi: 10.1016/j.ejphar.2011.06.027 21741965 PMC3169012

[4] McNeil BD Pundir P Meeker S Han L Undem BJ Kulka M . Identification of a mast-cell-specific receptor crucial for pseudo-allergic drug reactions. Nature (2015) 519:237–41. doi: 10.1038/nature14022 PMC4359082 25517090

[5] Gaudenzio N Sibilano R Marichal T Starkl P Reber LL Cenac N . Different activation signals induce distinct mast cell degranulation strategies. J Clin Invest (2016) 126:3981–98. doi: 10.1172/JCI85538 PMC5096814 27643442

[6] Azimi E Reddy VB Shade K-TC Anthony RM Talbot S Pereira PJS . Dual action of neurokinin-1 antagonists on mas-related GPCRs. JCI Insight (2016) 1. doi: 10.1172/jci.insight.89362 PMC5053144 27734033

[7] Elst J Maurer M Sabato V Faber MA Bridts CH Mertens C . Novel insights on MRGPRX2-mediated hypersensitivity to neuromuscular blocking agents and fluoroquinolones. Front Immunol (2021) 12:668962. doi: 10.3389/fimmu.2021.668962 34385999 PMC8353374

[8] Elst J Sabato V Faber M Bridts C Mertens C van Houdt M . MRGPRX2 and immediate drug hypersensitivity: Insights from cultured human mast cells. J Invest. Allergy Clin Immunol (2021) 31:489–99. doi: 10.18176/jiaci.0557 32732181

[9] Elst J Sabato V van der Poorten M-LM Faber M van Gasse AL de Puysseleyr LP . Peripheral blood cultured mast cells: Phenotypic and functional outcomes of different culture protocols. J Immunol Methods (2021) 492:113003. doi: 10.1016/j.jim.2021.113003 33647250

[10] Liu R Hu S Zhang Y Che D Cao J Wang J . Mast cell-mediated hypersensitivity to fluoroquinolone is MRGPRX2 dependent. Int Immunopharmacol. (2019) 70:417–27. doi: 10.1016/j.intimp.2019.02.001 30856392

[11] van Gasse AL Sabato V Uyttebroek AP Elst J Faber MA Hagendorens MM . Immediate moxifloxacin hypersensitivity: Is there more than currently meets the eye? Allergy (2017) 72:2039–43. doi: 10.1111/all.13236 28658502

[12] Lansu K Karpiak J Liu J Huang X-P McCorvy JD Kroeze WK . In silico design of novel probes for the atypical opioid receptor MRGPRX2. Nat Chem Biol (2017) 13:529–36. doi: 10.1038/nchembio.2334 PMC5391270 28288109

[13] Azimi E Reddy VB Lerner EA . MRGPRX2, atopic dermatitis, and red man syndrome. Itch (2017) 2:e5–5. doi: 10.1097/itx.0000000000000005 PMC5375112 28367504

[14] Zhang T Che D Liu R Han S Wang N Zhan Y . Typical antimicrobials induce mast cell degranulation and anaphylactoid reactions *via* MRGPRX2 and its murine homologue MRGPRB2. Eur J Immunol (2017) 47:1949–58. doi: 10.1002/eji.201746951 28688196

[15] Ebo DG Elst J Moonen N van der Poorten MM van Gasse AL Garvey LH . Mast cell activation test: A new asset in the investigation of the chlorhexidine cross-sensitization profile. Clin Exp Allergy (2022). doi: 10.1111/cea.14129 35305051

[16] Elst J van der Poorten MM van Gasse AL de Puysseleyr L Hagendorens MM Faber MA . Mast cell activation tests by flow cytometry: A new diagnostic asset? Clin Exp Allergy (2021) 51:1482–500. doi: 10.1111/cea.13984 34233046

[17] Elst J van der Poorten MM van Gasse AL Mertens C Hagendorens MM Ebo DG . Tryptase release does not discriminate between IgE- and MRGPRX2-mediated activation in human mast cells. Clin Exp Allergy (2022). doi: 10.1111/cea.14110 35152504

[18] Wedi B Gehring M Kapp A . The pseudoallergen receptor MRGPRX2 on peripheral blood basophils and eosinophils: Expression and function. Allergy (2020) 75:2229–42. doi: 10.1111/all.14213 32003863

[19] Sabato V van Gasse A Cop N Claesen K Decuyper II Faber MA . The mas-related G protein-coupled receptor MRGPRX2 is expressed on human basophils and up-regulated upon activation. J Allergy Clin Immunol (2017) 139:AB168. doi: 10.1016/j.jaci.2016.12.550

[20] Elst J Sabato V Mertens C Garvey LH Ebo DG . Association between mutated mas-related G protein-coupled receptor-X2 and rocuronium-induced intraoperative anaphylaxis. Comment Br J Anaesth. (2020) 125:e446–8. doi: 10.1016/j.bja.2020.08.035 32622467

[21] Shtessel M Limjunyawong N Oliver ET Chichester K Gao L Dong X . MRGPRX2 activation causes increased skin reactivity in patients with chronic spontaneous urticaria. J Invest Dermatol (2021) 141:678–681.e2. doi: 10.1016/j.jid.2020.06.030 32771471 PMC11658616

[22] Cop N Decuyper I Faber M Sabato V Bridts C Hagendorens M . Phenotypic and functional characterization of *in vitro* cultured human mast cells. Cytometry B Clin Cytom. (2017) 92:348–54. doi: 10.1002/cyto.b.21399 27401129

[23] Elst J Ebo DG Faber MA van Gasse AL Decuyper II van der Poorten M-LM . Culturing cells with mast cell phenotype and function: Comparison of peripheral blood and bone marrow as a source. J Immunol Methods (2021) 495:113061. doi: 10.1016/j.jim.2021.113061 33933470

[24] Erdmann SM Sachs B Schmidt A Merk HF Scheiner O Moll-Slodowy S . *In vitro* analysis of birch-Pollen-Associated food allergy by use of recombinant allergens in the basophil activation test. Int Arch Allergy Immunol (2005) 136:230–8. doi: 10.1159/000083949 15713985

[25] de Jonge HJM Fehrmann RSN de Bont ESJM Hofstra RMW Gerbens F Kamps WA . Evidence based selection of housekeeping genes. PloS One (2007) 2:e898. doi: 10.1371/journal.pone.0000898 17878933 PMC1976390

[26] Karhu T Akiyama K Vuolteenaho O Bergmann U Naito T Tatemoto K . Mast cell degranulation *via* MRGPRX2 by isolated human albumin fragments. Biochim Biophys Acta (BBA) - Gen Subj (2017) 1861:2530–4. doi: 10.1016/j.bbagen.2017.08.013 28844982

[27] Subramanian H Gupta K Ali H . Roles of mas-related G protein–coupled receptor X2 on mast cell–mediated host defense, pseudoallergic drug reactions, and chronic inflammatory diseases. J Allergy Clin Immunol (2016) 138:700–10. doi: 10.1016/j.jaci.2016.04.051 PMC5014572 27448446

[28] van Gasse AL Elst J Bridts CH Mertens C Faber M Hagendorens MM . Rocuronium hypersensitivity: Does off-target occupation of the MRGPRX2 receptor play a role? J Allergy Clin Immunol Pract (2019) 7:998–1003. doi: 10.1016/j.jaip.2018.09.034 30315997

[29] Leysen J de Witte L Sabato V Faber M Hagendorens M Bridts C . IgE-mediated allergy to pholcodine and cross-reactivity to neuromuscular blocking agents: Lessons from flow cytometry. Cytometry B Clin Cytom. (2013) 84B:65–70. doi: 10.1002/cyto.b.21074 23355309

[30] van Gasse AL Hagendorens MM Sabato V Bridts CH de Clerck LS Ebo DG . IgE to poppy seed and morphine are not useful tools to diagnose opiate allergy. J Allergy Clin Immunol Pract (2015) 3:396–9. doi: 10.1016/j.jaip.2014.12.002 25956313

[31] van Gasse AL Sabato V Faber MA Hagendorens MM Ebo DG . An alternative explanation for immediate hypersensitivity reactions to opioids. J Allergy Clin Immunol Pract (2017) 5:1806. doi: 10.1016/j.jaip.2017.08.016 29122168

[32] Uyttebroek AP Sabato V Leysen J Bridts CH de Clerck LS Ebo DG . Flowcytometric diagnosis of atracurium-induced anaphylaxis. Allergy (2014) 69:1324–32. doi: 10.1111/all.12468 24961660

[33] Motakis E Guhl S Ishizu Y Itoh M Kawaji H de Hoon M . Redefinition of the human mast cell transcriptome by deep-CAGE sequencing. Blood (2014) 123:e58–67. doi: 10.1182/blood-2013-02-483792 PMC3999759 24671954

[34] MacGlashan D Xia HZ Schwartz LB Gong J . IgE-regulated loss, not IgE-regulated synthesis, controls expression of FcepsilonRI in human basophils. J Leukoc Biol (2001) 70:207–18. doi: 10.1189/jlb.70.2.207 11493612

[35] Ebo DG Bridts CH Hagendorens MM Aerts NE de Clerck LS Stevens WJ . Basophil activation test by flow cytometry: Present and future applications in allergology. Cytometry B Clin Cytom. (2008) 74B:201–10. doi: 10.1002/cyto.b.20419 18412216

[36] Knol EF Koenderman L Mul FPJ Verhoeven AJ Roos D . Differential activation of human basophils by anti-IgE and formyl-methionyl-leucylphenylalanine. indications for protein kinase c-dependent and -independent activation pathways. Eur J Immunol (1991) 21:881–5. doi: 10.1002/eji.1830210404 1708341

[37] Guhl S Babina M Neou A Zuberbier T Artuc M . Mast cell lines HMC-1 and LAD2 in comparison with mature human skin mast cells - drastically reduced levels of tryptase and chymase in mast cell lines. Exp Dermatol (2010) 19:845–7. doi: 10.1111/j.1600-0625.2010.01103.x 20545757

[38] Chompunud Na Ayudhya C Roy S Alkanfari I Ganguly A Ali H . Identification of gain and loss of function missense variants in MRGPRX2’s transmembrane and intracellular domains for mast cell activation by substance p. Int J Mol Sci (2019) 20:5247. doi: 10.3390/ijms20215247 31652731 PMC6862462

[39] Babina M Wang Z Li Z Franke K Guhl S Artuc M . FcϵRI- and MRGPRX2-evoked acute degranulation responses are fully additive in human skin mast cells. Allergy (2022). doi: 10.1111/all.15270 35246987

[40] Yang S Liu Y Lin AA Cavalli-Sforza LL Zhao Z Su B . Adaptive evolution of MRGX2, a human sensory neuron specific gene involved in nociception. Gene (2005) 352:30–5. doi: 10.1016/j.gene.2005.03.001 15862286

[41] Reddy VB Graham TA Azimi E Lerner EA . A single amino acid in MRGPRX2 necessary for binding and activation by pruritogens. J Allergy Clin Immunol (2017) 140:1726–8. doi: 10.1016/j.jaci.2017.05.046 PMC5723232 28689793

[42] Porebski G Kwiecien K Pawica M Kwitniewski M . Mas-related G protein-coupled receptor-X2 (MRGPRX2) in drug hypersensitivity reactions. Front Immunol (2018) 9:3027. doi: 10.3389/fimmu.2018.03027 30619367 PMC6306423

[43] Wang Z Franke K Bal G Li Z Zuberbier T Babina M . MRGPRX2-mediated degranulation of human skin mast cells requires the operation of gαi, gαq, ca++ channels, ERK1/2 and PI3K–interconnection between early and late signaling. Cells (2022) 11:953. doi: 10.3390/cells11060953 35326404 PMC8946553

[44] Zhang F Hong F Wang L Fu R Qi J Yu B . MrgprX2 regulates mast cell degranulation through PI3K/AKT and PLCγ signaling in pseudo-allergic reactions. Int Immunopharmacol. (2022) 102:108389. doi: 10.1016/j.intimp.2021.108389 34920312

[45] Matsuo K Fujiwara K Omuro N Kimura I Kobayashi K Yoshio T . Effects of the fluoroquinolone antibacterial drug ciprofloxacin on ventricular repolarization in the halothane-anesthetized Guinea pig. J Pharmacol Sci (2013) 122:205–12. doi: 10.1254/jphs.13020FP 23803533

[46] Bito M Tomita T Komori M Taogoshi T Kimura Y Kihira K . The mechanisms of insulin secretion and calcium signaling in pancreatic β-cells exposed to fluoroquinolones. Biol Pharm Bull (2013) 36:31–5. doi: 10.1248/bpb.b12-00425 23302634

[47] Fujisawa D Kashiwakura J Kita H Kikukawa Y Fujitani Y Sasaki-Sakamoto T . Expression of mas-related gene X2 on mast cells is upregulated in the skin of patients with severe chronic urticaria. J Allergy Clin Immunol (2014) 134:622–633.e9. doi: 10.1016/j.jaci.2014.05.004 24954276

[48] Kurimoto Y de Weck AL Dahinden CA . The effect of interleukin 3 upon IgE-dependent and IgE-independent basophil degranulation and leukotriene generation. Eur J Immunol (1991) 21:361–8. doi: 10.1002/eji.1830210217 1705512

