# Supplementary material for: Mas-related G protein-coupled receptor MRGPRX2 in human basophils: Expression and functional studies
Source: Front Immunol. 2023 Jan 16;13:1026304. doi: 10.3389/fimmu.2022.1026304 (PMC9885256; doi:10.3389/fimmu.2022.1026304)
Supplement: Supplementary file 1 [file DataSheet_1.docx]

Supplementary Material

Mas-related G protein-coupled receptor MRGPRX2 in human basophils: expression and functional studies

Alessandro Toscano, Jessy Elst, Athina L. Van Gasse, Michiel Beyens, Marie-Line van der Poorten, Chris H. Bridts, Christel Mertens, Michel Van Houdt, Margo M. Hagendorens, Samuel Van Remoortel, Jean-Pierre Timmermans, Didier G. Ebo*, Vito Sabato.

*** Correspondence:** Didier G. Ebo: immuno@uantwerpen.be

## Supplementary Figures

##
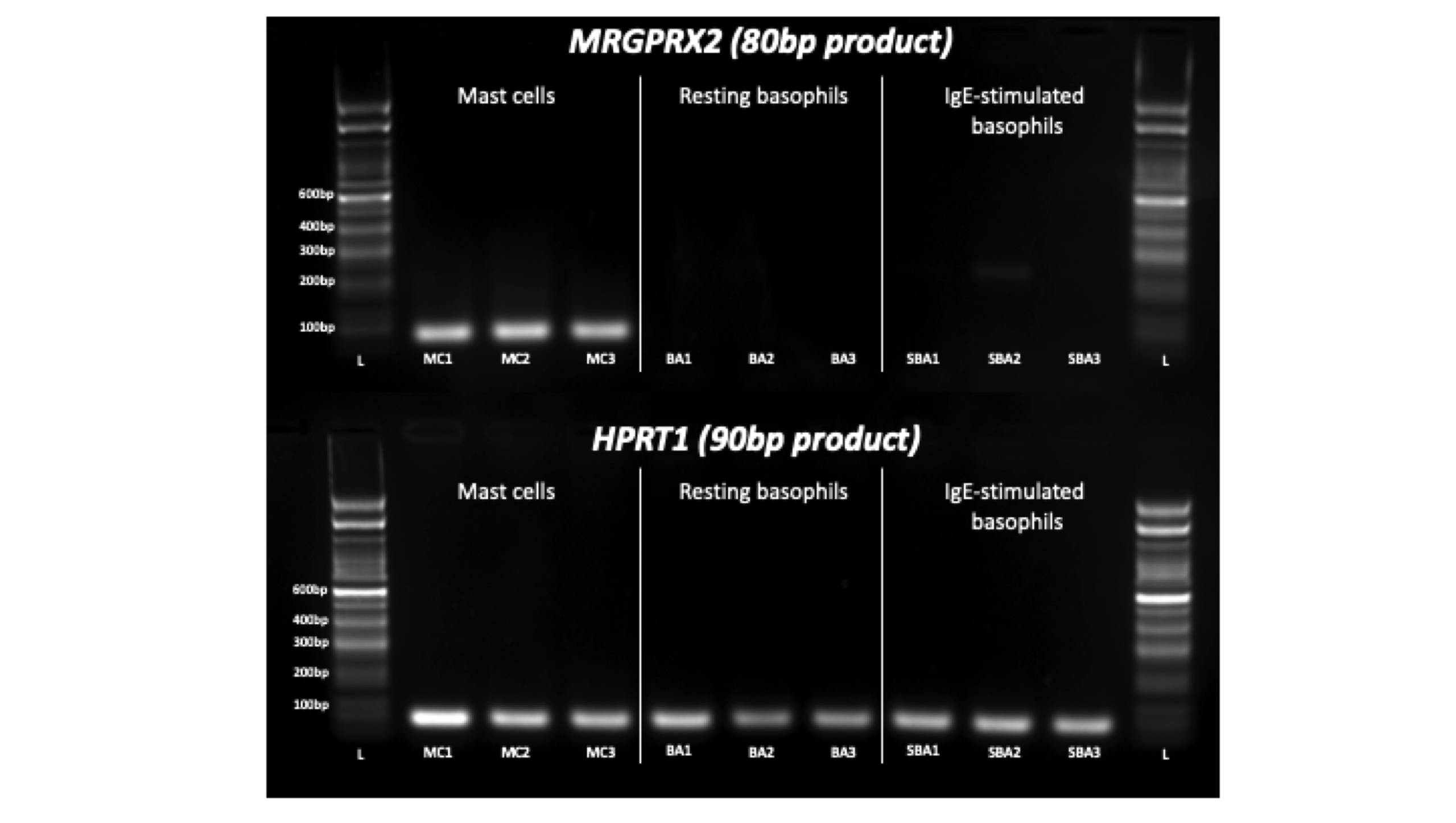


**Supplementary Figure 1.** **Expression of MRGPRX2 mRNA in peripheral blood cultured mast cells (PBCMCs) and whole blood basophils.** Gel electrophoresis of the qPCR products of MRGPRX2 (upper part) and HPRT1 (lower part) in mast cells (MC1, MC2, MC3), resting basophils (BA1, BA2, BA3) and anti-IgE-stimulated basophils (SBA1, SBA2, SBA3). Expected band size MRGPRX2: 80bp; Expected band size HPRT1: 90bp; L: Ladder; n = 3 independent samples per group.


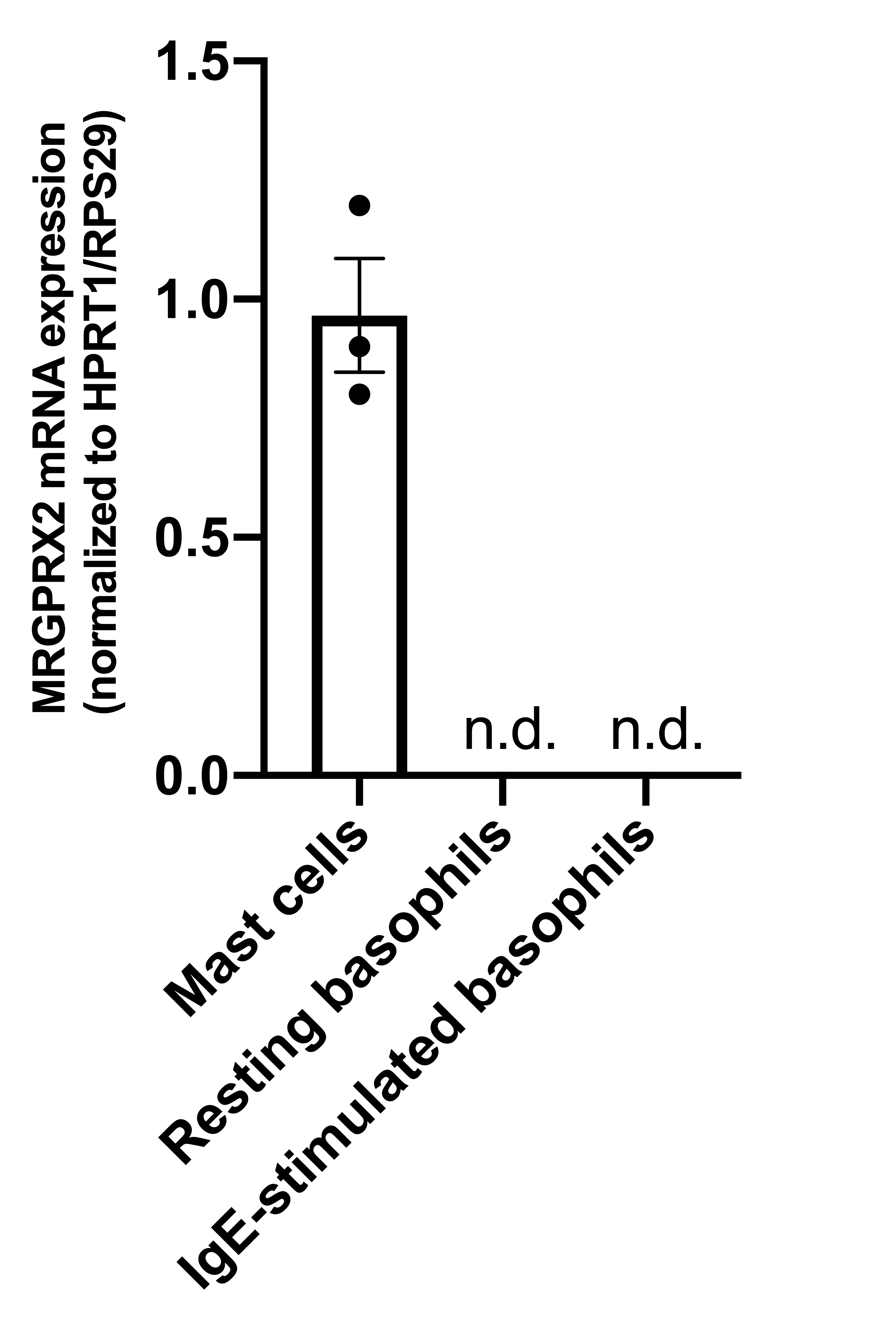


**Supplementary Figure 2. MRGPRX2 mRNA expression after normalization against HRPRT1 and RPS29 in mast cells and basophils.** n.d. = not detected.

##
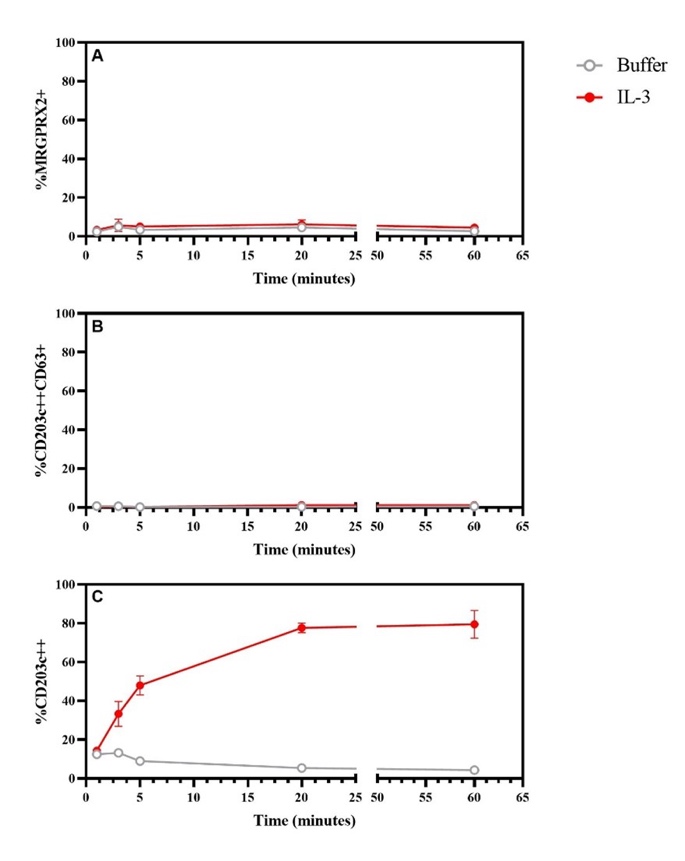


## Supplementary Figure 3. Time kinetic of the effect of IL-3 on whole blood basophils. Time curves for MRGPRX2 (A), CD63 (B), and CD203c (C) membrane expression of whole blood basophils from HCs after incubation with IL-3 (10 ng/mL) (n=4). HCs = healthy controls.


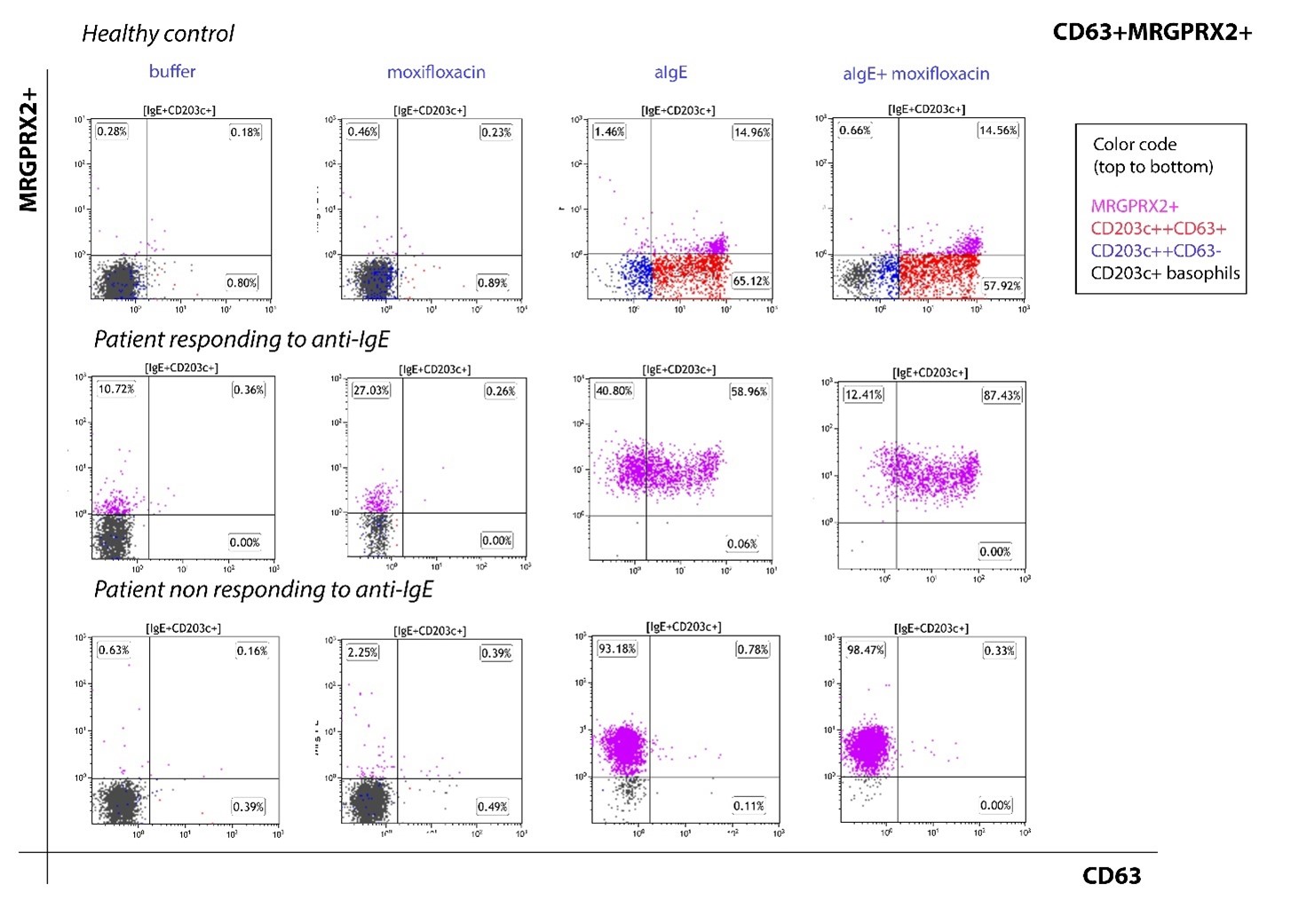


**Supplementary Figure 4. Co-incubation experiments with anti-IgE and moxifloxacin: representative experiments.** Representative plots on the effect of anti-IgE, moxifloxacin 0.025 mmol/L and anti-IgE in co-incubation with moxifloxacin 0.025 mmol/L on MRGPRX2, CD63 and CD203c membrane expression of basophils from a CD63-responding HC, a CD63-responding MOX, and a CD63-non-responding MOX (analyses performed at 5 minutes). MOX= patient with immediate type hypersensitivity to moxifloxacin; HC= healthy control.


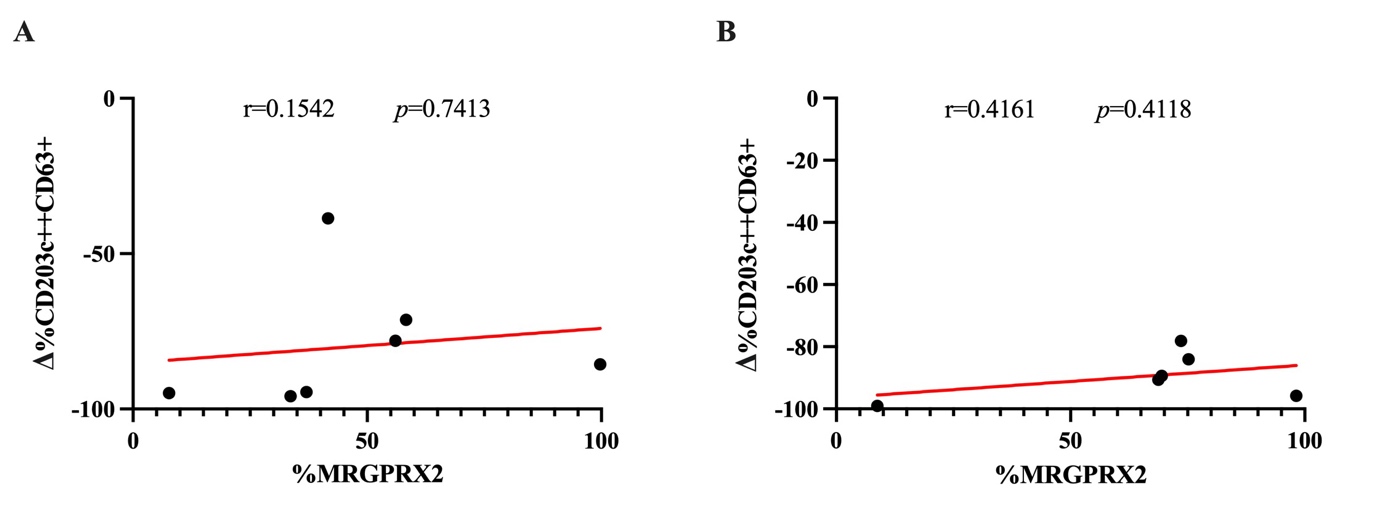


**Supplementary figure 5.** **Correlation between MRGPRX2 expression and inhibitory effect on degranulation of co-incubation of whole blood basophils with moxifloxacin 2.5 mmol/L and anti-IgE vs. anti-IgE alone after 3 minutes.** (A) CD63-responding MOXs (n=7); (B) CD63-responding HCs (n=6). MOXs = patients with immediate type hypersensitivity to moxifloxacin; HCs = healthy controls.


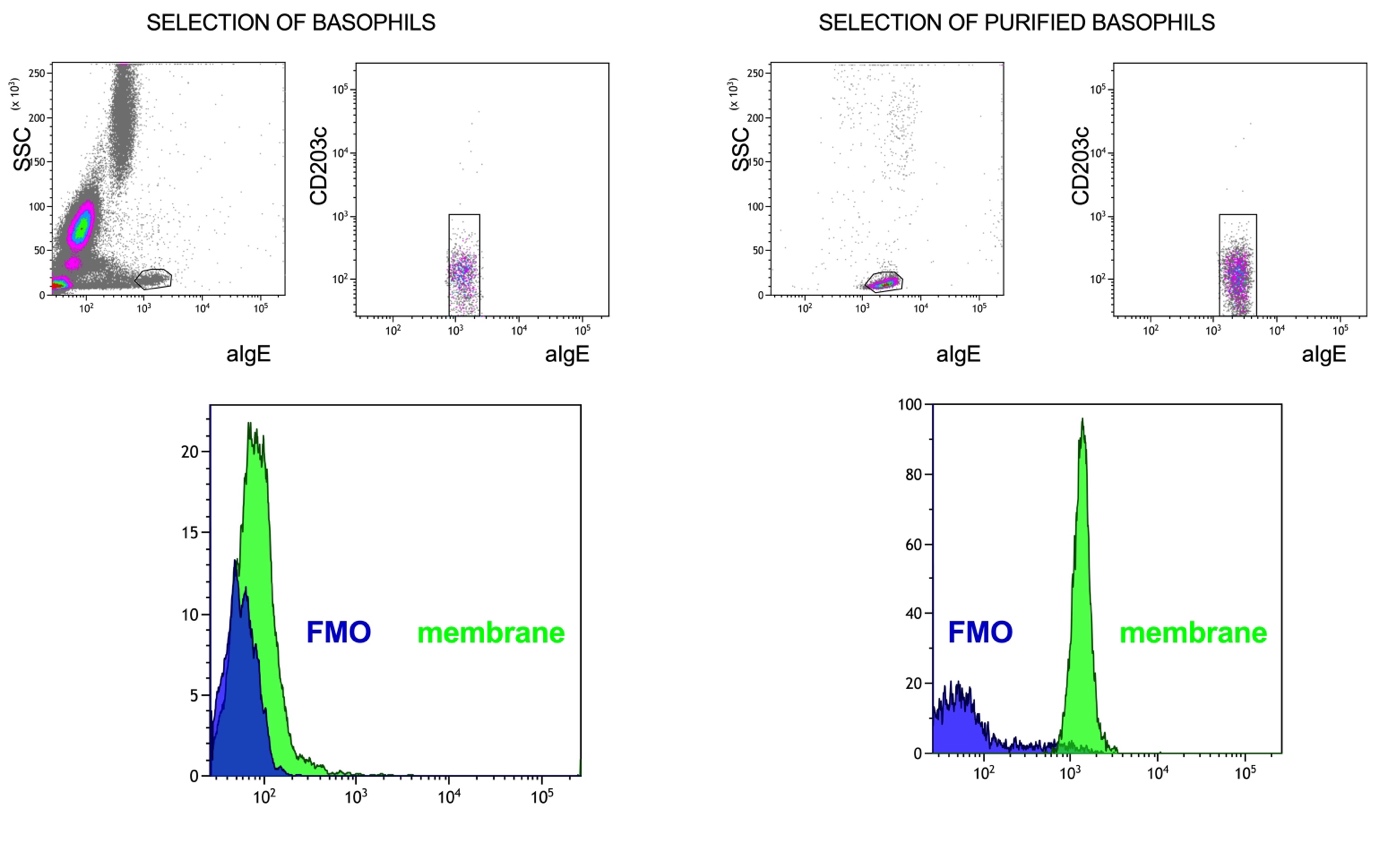


**Supplementary Figure 6.** **Membrane MRGPRX2 expression of whole blood and purified basophils.** Representative plots of membrane (green histogram) MRGPRX2 expression on whole blood and purified basophils from one HC. FMO = fluorescence minus one sample (blue histogram).


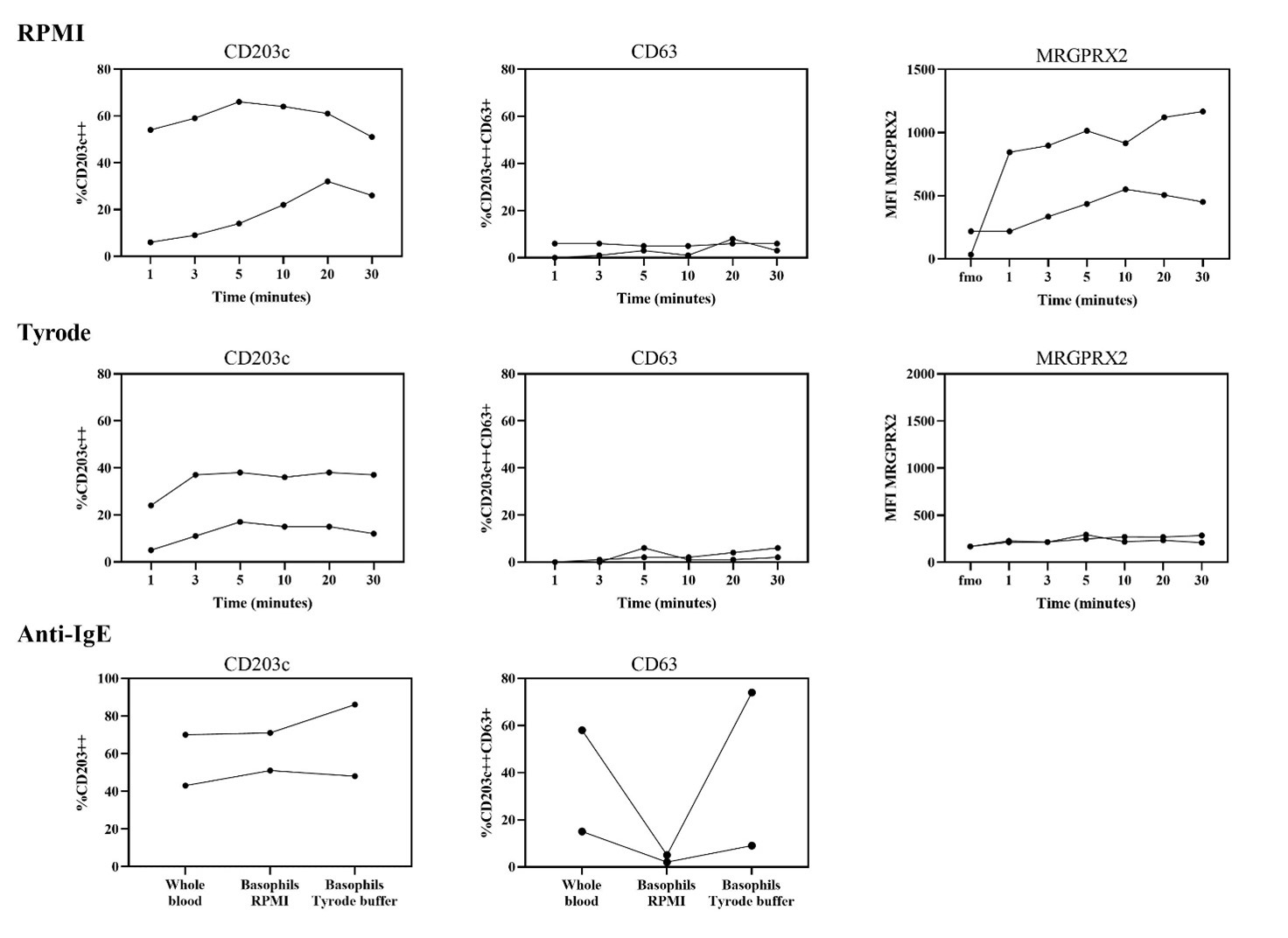


**Supplementary Figure 7**. **Effect of resuspending medium on purified basophils.** CD203c, CD63, and MRGPRX2 (expressed as mean fluorescence intensity (MFI)) expression on the surface membrane of purified basophils from HCs after resuspension in RPMI medium or Tyrode medium and expression of CD203c and CD63 on purified basophils from the same HCs after resuspension in RPMI medium or Tyrode medium and incubation with anti-IgE (n=2).


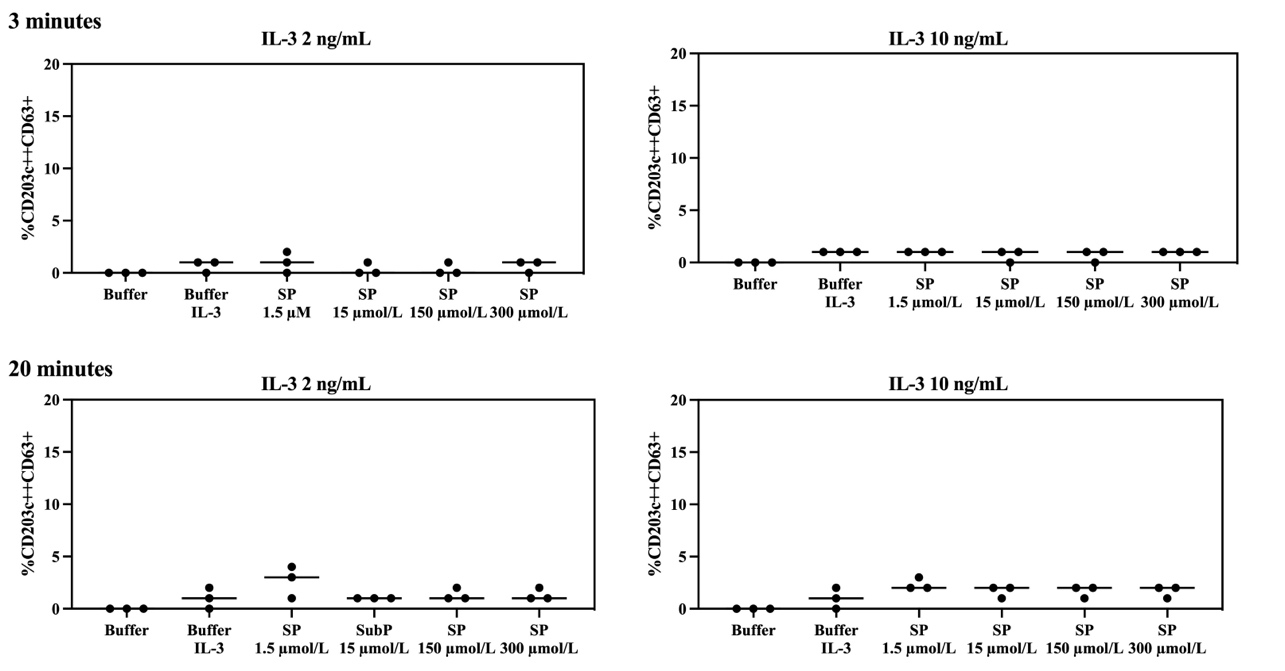


**Supplementary Figure 8. Effect of SP on whole blood basophils primed with IL-3.**

Effect of incubation of SP on CD63 expression on the surface membrane of whole blood basophils from HCs primed with two different concentrations of IL-3 (2 ng/mL and 10 ng/mL) (analyses performed after 3 and 20 minutes from the priming) (n=3). SP = substance P.


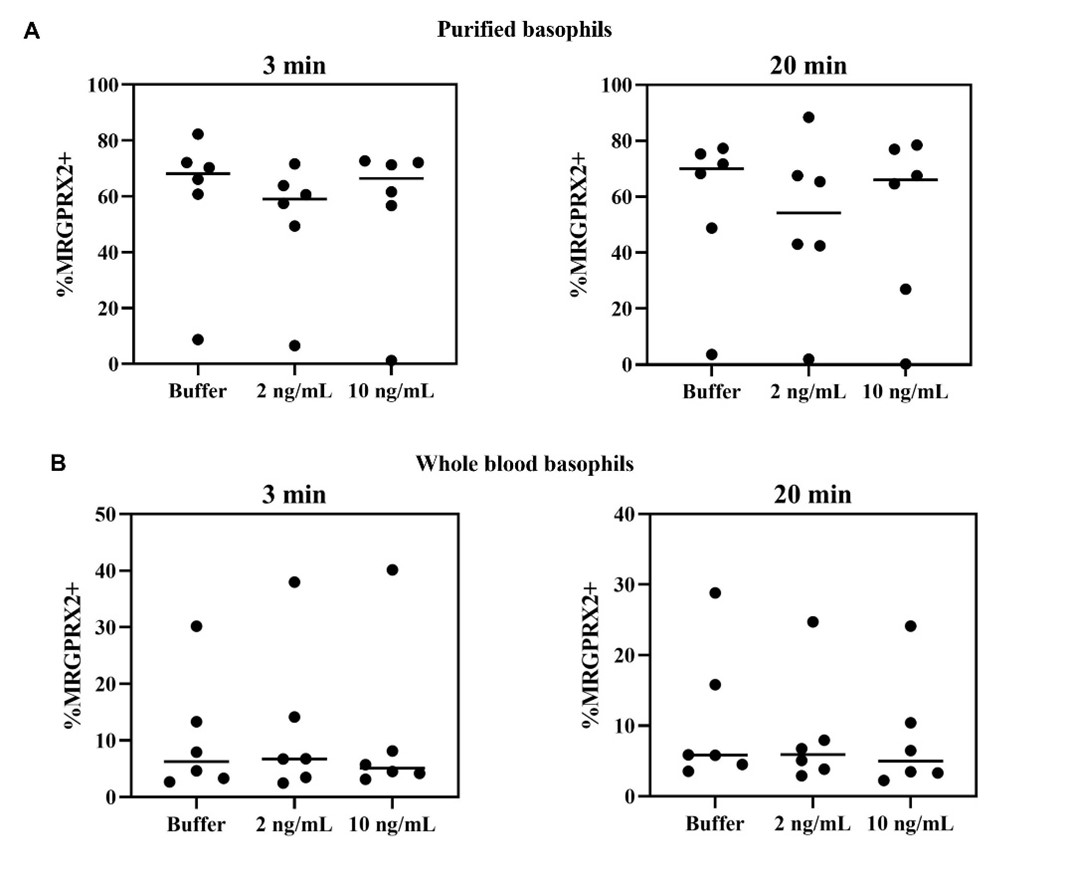


**Supplementary Figure 9. MRGPRX2 expression in purified and whole blood basophils primed with IL-3.**

Effect of priming with two different concentrations of IL-3 (2 ng/mL and 10 ng/mL) on MRGPRX2 expression on the surface membrane of purified basophils resuspended in RPMI medium (A) and whole blood basophils (B) from HCs (analyses performed after 3 and 20 minutes from the priming) (n=6). HCs = healthy controls.


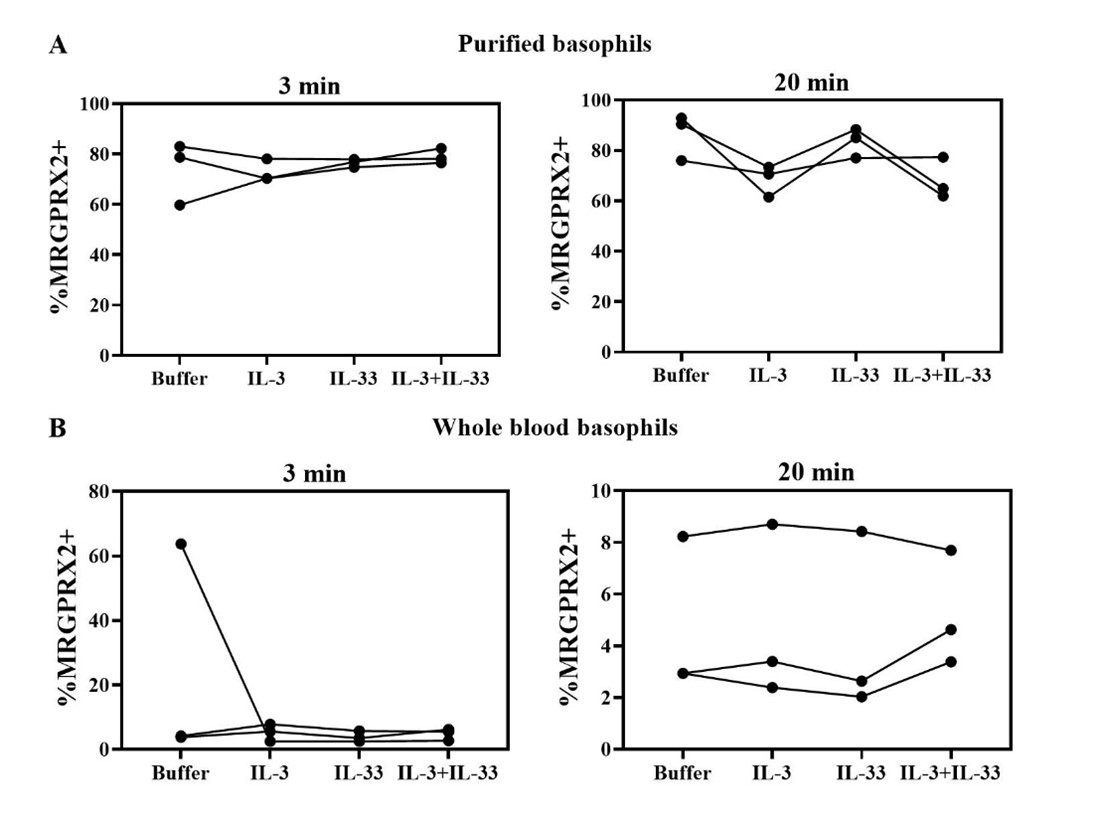


**Supplementary Figure 10.** **MRGPRX2 expression in purified and whole blood basophils incubated with IL-3, IL-33, or both.** Effect of incubation with IL-3 (10 ng/mL), IL-33 (30 ng/mL), or both on MRGPRX2 expression on the surface membrane of purified basophils resuspended in RPMI medium (A) and whole blood basophils (B) from HCs after 3 and 20 minutes (n=3). HCs = healthy controls.
